# Subendothelial stiffness alters endothelial cell traction force generation while exerting a minimal effect on the transcriptome

**DOI:** 10.1038/s41598-019-54336-2

**Published:** 2019-12-03

**Authors:** Effie E. Bastounis, Yi-Ting Yeh, Julie A. Theriot

**Affiliations:** 1 Department of Biology and Howard Hughes Medical Institute, University of Washington, Seattle, WA 98195-1800 USA; 2 Department of Bioengineering, University of California San Diego, La Jolla, California USA

**Keywords:** Mechanotransduction, Actin

## Abstract

Endothelial cells respond to changes in subendothelial stiffness by altering their migration and mechanics, but whether those responses are due to transcriptional reprogramming remains largely unknown. We measured traction force generation and also performed gene expression profiling for two endothelial cell types grown in monolayers on soft or stiff matrices: primary human umbilical vein endothelial cells (HUVEC) and immortalized human microvascular endothelial cells (HMEC-1). Both cell types respond to changes in subendothelial stiffness by increasing the traction stresses they exert on stiffer as compared to softer matrices, and exhibit a range of altered protein phosphorylation or protein conformational changes previously implicated in mechanotransduction. However, the transcriptome has only a minimal role in this conserved biomechanical response. Only few genes were differentially expressed in each cell type in a stiffness-dependent manner, and none were shared between them. In contrast, thousands of genes were differentially regulated in HUVEC as compared to HMEC-1. HUVEC (but not HMEC-1) upregulate expression of TGF-β2 on stiffer matrices, and also respond to application of exogenous TGF-β2 by enhancing their endogenous TGF-β2 expression and their cell-matrix traction stresses. Altogether, these findings provide insights into the relationship between subendothelial stiffness, endothelial mechanics and variation of the endothelial cell transcriptome, and reveal that subendothelial stiffness, while critically altering endothelial cells’ mechanical behavior, minimally affects their transcriptome.

## Introduction

Subendothelial stiffening often occurs with aging and in multiple pathologies such as atherosclerosis and hypertension constituting a risk factor for development of cardiovascular disease^1,2^. Endothelial cells (ECs) that form a single monolayer *in vivo* to line the inner lumen of blood vessels, respond to changes in the mechanics of their extracellular matrix (ECM), such as its stiffness, by changing their migration, proliferation and barrier integrity, thus contributing to the emergence of these pathologies^3–5^. Understanding the interplay between the micro-environmental mechanical determinants and EC behavior is therefore pertinent to understanding vascular biology and might have important therapeutic implications.

ECs exhibit remarkable phenotypic heterogeneity, and the basis of these morphological, molecular and functional differences is still not completely characterized^6,7^. It has been previously proposed that the spatiotemporal differences in chemical and also mechanical cues relayed to ECs by their environment theoretically could be sufficient to explain their structural and functional differences^8^. Examples of mechanical signals relayed to ECs include subendothelial stiffness, fluid shear flow and mechanical strains. However, even when ECs from different anatomical locations are placed *in vitro* in the same biomechanical environment, they can still display a unique behavior intrinsic to the ECs themselves and not determined by differential culture or microenvironmental conditions^9–11^. For instance, the response of human umbilical cord endothelial cells (HUVEC) to changes in curvature or shear stress applied in tissue culture is completely distinct from that of brain microvascular ECs^9^.

Transcriptomic profiling has advanced our understanding of how differential gene expression is linked to altered cell behavior. Specifically, it has provided insight into the complex biological pathways and molecular mechanisms that regulate changes in cellular behavior in response to mechanical cues for certain cells types, such as mesenchymal stem cells, vascular smooth muscle cells and certain endothelial cell types, all of which were found to be extremely sensitive to substrate stiffness^12–17^. However, in most of these studies cell confluency was either low or not explicitly stated. Cell density plays a crucial role in the response of ECs to mechanical cues and in the forces transduced by ECs on their ECM and on each other^18,19^ and increased cell density can even override the effect of ECM stiffness in certain cell types^20^. Inspired by these studies, we sought to answer two important previously unexplored questions: (1) Are the biomechanical changes in response to subendothelial stiffness observed for ECs in monolayers due to transcriptional regulation of key stiffness-sensitive genes? and (2) Is the transcriptomic profile of ECs in monolayers dominated by the specific EC type or by the mechanical microenvironment, in particular subendothelial stiffness?

In this study, we compared the responses of two different types of ECs to growth on stiff versus soft hydrogel substrates, primary human umbilical vein endothelial cells (HUVEC) cultured from normal human tissue and immortalized human microvascular endothelial cells (HMEC-1) that were transformed using SV40 large T antigen^21^. Both cell types in confluent monolayers changed their mechanical behavior in response to increasing subendothelial stiffness similarly, by elevating their cell-matrix traction stresses on stiffer as compared to softer matrices, and altering protein phosphorylation profiles associated with mechanotransduction. However only very modest stiffness-dependent alterations in gene expression were observed using RNA sequencing.

## Results

### ECs in monolayers exert increased cell-matrix traction stresses when residing on stiff as compared to soft hydrogels

To assess how subendothelial stiffness affects EC mechanics and how that is related to changes in the endothelial transcriptome, we cultured ECs as confluent monolayers on substrates of varying stiffness. Trying to mimic (patho)physiologic subendothelial stiffness, we built collagen-I coated soft hydrogels of 3 kPa (lower range of EC basement membrane reported stiffness where cells can still in *vitro* form monolayers) and stiffer hydrogels of either 35 kPa or 70 kPa (higher ranges of basement membrane reported stiffness often associated with aging or pathologies)^22–24^. The resulting stiffness of the gels was confirmed through atomic force microscopy (Supplementary Fig. S1a). Hydrogels were embedded with fluorescent beads such that when ECs establish their focal adhesions and start pulling on the hydrogels and on each other, we can use time-lapse microscopy to monitor the cell and bead movement. Using the fluorescence images of the beads we then infer the deformations the cells impart and the stresses they exert on their matrix through traction force microscopy (TFM)^25^. 3 kPa is the lowest stiffness we examined since as previously reported, we find that ECs seeded on <3 kPa stiffness gels do not consistently form monolayers but rather vessel-like patterns with intermittent gaps^26,27^. 70 kPa gels are considered as our “STIFF” condition given previous reported values of pathological subendothelial stiffness measured in clinical studies^22–24^. However, for TFM only we used as “STIFF” 35 kPa gels since this was found to be the highest stiffness on which the cells can still deform the gels.

**Figure 1:**
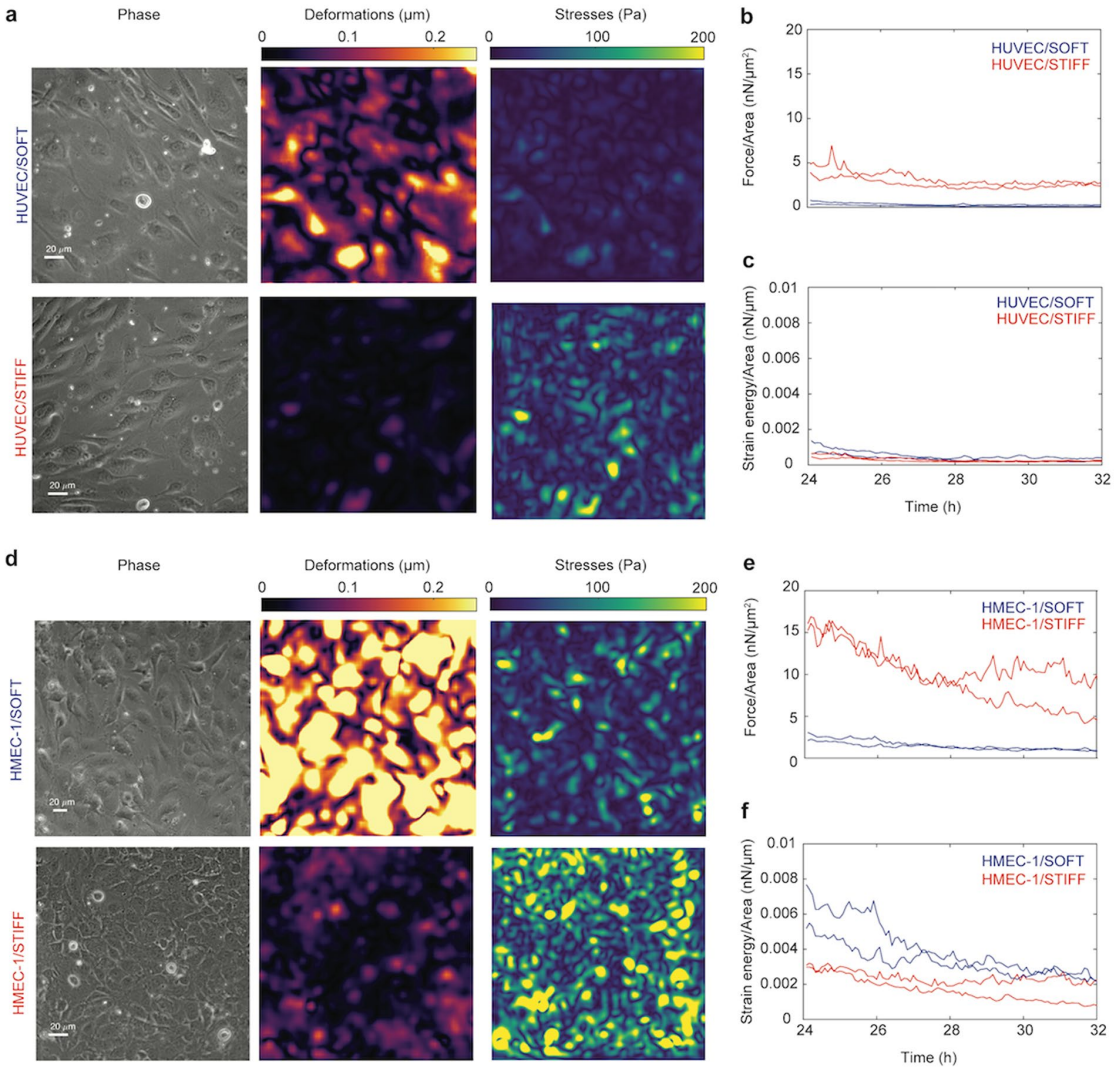
Endothelial cells in monolayers exert higher cell-matrix traction forces onto stiff as compared to soft hydrogels. (**a**) Representative phase contrast images (phase, first column) and cell-matrix deformation maps (second column, color indicates deformation magnitude in μm) and traction stresses (third column, color indicates stress magnitude in Pa) exerted by confluent HUVEC adherent onto soft 3 kPa or stiff 35 kPa hydrogels. (**b,c**) Time evolution of the integral of the traction force magnitude over the whole field of view to its area (nN/μm^2^) (**b**) and of the total strain energy imparted by the cells per area of field of view (nN/μm) (**c**) calculated for two different regions within confluent HUVEC monolayers for cells residing on soft 3 kPa (blue) or stiff 35 kPa (red) matrices. (**d–f**) Same as in panels a-c but corresponding to HMEC-1 monolayers.

By performing TFM, we found that both HUVEC and HMEC-1 followed via time-lapse microscopy between 24 h to 32 h post-seeding generate smaller deformations on stiffer 35 kPa gels as opposed to the softer 3 kPa, and overall HMEC-1 generate larger deformations than HUVEC under similar conditions (Fig. 1). Interestingly, for both cell types the traction stresses they exert and the total traction force magnitude over the field of view is significantly higher for ECs residing on 35 kPa gels as compared to 3 kPa gels (Fig. 1a,b,d,e). This finding suggests that the internal cytoskeleton and contractility of these two EC types changes depending on the mechanical properties of their subendothelium, in a way that might be similar in both EC types. Note that the strain energy, which is the mechanical work spent by the cells to deform their matrix, for both cell types is similar irrespective of substrate stiffness (Fig. 1c,f).

**Figure 2:**
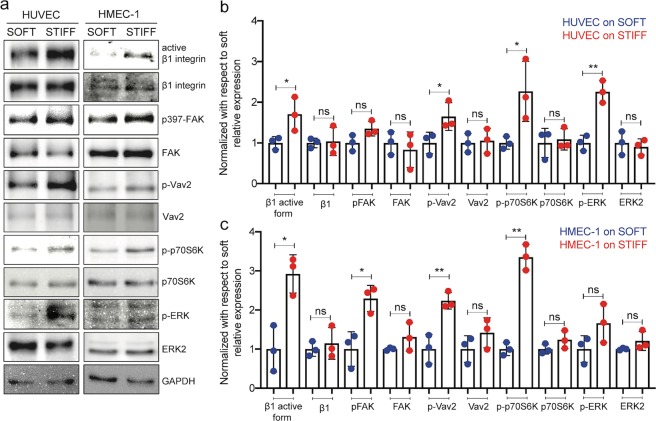
Post-transcriptional changes on ECs in monolayers grown on soft versus stiff hydrogels. (**a**) Western blots from whole HUVEC or HMEC-1 lysates of cells previously residing on soft gels (3 kPa) or stiff gels (70 kPa). Representative cropped blots are displayed and full-length blots can be found in the supplementary material (Supplementary Fig. S2). Each row shows a different protein whose expression, phosphorylation or conformation was probed, namely: active form of integrin β1, total integrin β1, p397FAK, total FAK, p-Vav2, total Vav2, p-p70S6K, total p70S6K, p-ERK, total ERK and GAPDH (used as loading control). Experiments were performed N = 3 times. (**b**) Bar plots show relative expression of the proteins probed in panel a for HUVEC cells residing on soft (blue) or stiff (red) gels. All measurements were normalized to GAPDH expression for each condition, and expressed as fold-change relative to the median expression level on soft substrates. One or two asterisks denote statistically significant differences between the medians of two distributions (<0.05 or <0.01 respectively; unpaired t-test) and non-significant differences are denoted as ns. (**c**) Same as panel b but for HMEC-1.

Given that previous studies have shown that EC protein levels can be sensitive to subendothelial stiffness^28,29^, we sought to evaluate whether in our system protein expression or activity would be modulated by matrix stiffness under our experimental conditions. We thus performed Western blot analysis for a number of EC proteins whose activity or conformation has been previously shown to depend on subendothelial stiffness^28–32^. To that end we measured protein levels for the active form of integrin β1 and integrin β1^30^, p397FAK and FAK^32^, p-Vav2 and Vav2^28^, p-p70S6K and p70S6K^31^, p-ERK and ERK^5^. We found that the phosphorylation level or conformation of all these proteins changes for one or both cell types (Fig. 2; Supplementary Fig. S2). For both cell types the protein level of the active form of integrin β1, but not total integrin β1 expression, is increased for cells residing on stiff as compared to soft substrates (Fig. 2a–c). Similarly, pVav2 and p-p70S6K, but not total protein levels of Vav2 and p70S6K, are increased for both cell types on stiff as opposed to soft matrices. ERK2 protein levels remain constant while phosphorylation increases with stiffness for HUVEC (but not HMEC-1). Finally, total FAK levels remain constant but FAK activity increases significantly for HMEC-1 only (but not HUVEC). These results demonstrate that known mechanosensitive proteins in these two EC types are responding as expected to changes in matrix stiffness, and that this response is largely mediated by changes in protein phosphorylation or conformation (Fig. 2b,c).

### Differentially expressed genes (DEGs) arise due to EC type rather than subendothelial stiffness

We sought to understand whether the subendothelial stiffness-dependent elevation of the EC traction stresses is additionally regulated by changes in the EC transcriptome. To this end, we implemented RNA sequencing to comprehensively define gene expression in HUVEC and HMEC-1 grown in monolayers and in similar densities, in response to physiologically soft (3 kPa, on SOFT) and pathologically stiff (70 kPa, on STIFF) matrices (Supplementary Fig. S1a,b). Due to inherent variability of biological samples and to increase our confidence in identification of DEGs, we decided to sequence 6 replicates per condition. Using GENCODE annotations (grch38)^33^, we identified a total of 15,797 genes expressed in HUVEC and 16,027 genes expressed in HMEC-1, after disregarding genes with base mean normalized count lower than 10^34^.

**Figure 3:**
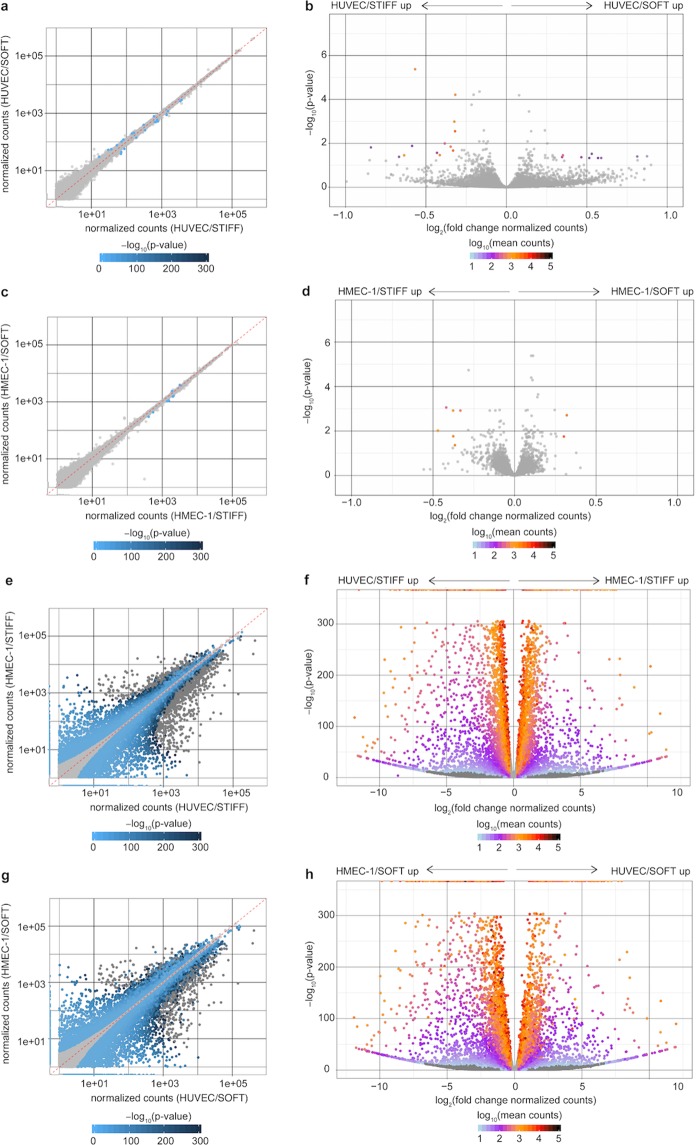
Endothelial origin but not matrix stiffness strongly determines the transcriptome of endothelial cells. (**a,c,e,g**) Scatter plots of expressed genes showing normalized counts of gene expression in the x and y axes for the indicated groups: (**a**) HUVEC on stiff 70 kPa (N = 6) versus soft 3 kPa matrices (N = 6), (**c**) HMEC-1 on stiff 70 kPa (N = 6) versus soft 3 kPa matrices (N = 6), and (**d**) HUVEC (N = 6) versus HMEC-1 on stiff 70 kPa matrices (N = 6) and (**g**) HUVEC (N = 6) versus HMEC-1 on soft 3 kPa matrices (N = 6). Light gray dots represent genes that are not differentially expressed while differentially expressed genes (DEGs) are shown as dots color-coded by their -log_10_ p-values. (**b,d,f,h**) Volcano plots showing DEGs between the same groups compared as above. The -log_10_ p-values (y-axis) are plotted against the average log_2_ fold changes in expression (x-axis). Non DEGs are plotted in light gray. DEGs are color-coded depending on the log_10_ of their mean normalized counts.

To our surprise, differential expression (DE) analysis of HUVEC on STIFF versus HUVEC on SOFT led us identify just 24 DEGs using typical thresholds (see Methods; Fig. 3a,b and Supplementary Fig. S3 and Table S1)^34^. When we then compared HMEC-1 on STIFF versus SOFT, we found just 8 DEGs (Fig. 3c,d and Supplementary Fig. S4 and Table S2). In contrast, comparison of HUVEC versus HMEC-1 on STIFF revealed a total of 10020 DEGs (Fig. 3e,f and Supplementary Fig. S5 and Table S4) while comparison of HUVEC versus HMEC-1 on SOFT revealed 10162 DEGs (Fig. 2g,h and Supplementary Fig. S6 and Table S4). Of those, 44 genes were solely expressed in HMEC-1 and 147 solely in HUVEC (Supplementary Table S5). Note that for the above we used a low threshold of at least 1.23-fold change. If a threshold of 2-fold change was applied, there would be no stiffness-dependent DEGs identified for either EC type, while HUVEC versus HMEC-1 on STIFF would yield 4449 DEGs and HUVEC versus HMEC-1 on SOFT 4558 DEGs. These results suggest that the specific EC type profoundly defines the transcriptome of ECs, and that matrix stiffness has only a minimal effect, at least for these two specific EC types studied and under the conditions where we examined them (*i.e*. confluent monolayers, with cells seeded for 24 h on collagen I-coated gels). In addition, we discovered that HUVEC show more stiffness-sensitive DEGs as compared to HMEC-1, and that none of these few identified DEGs are shared in common between the two EC types.

**Figure 4:**
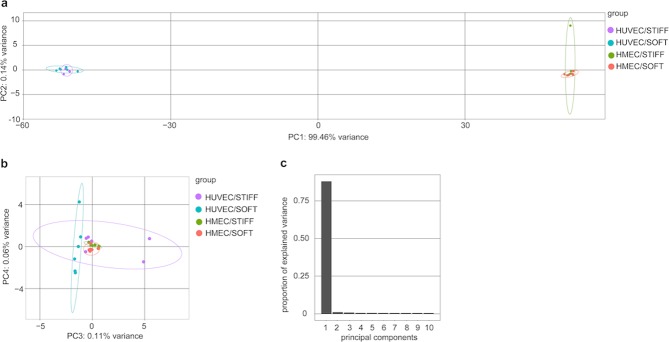
Principal component analysis (PCA) confirms that endothelial origin but not matrix stiffness is a major contributor of expression differences. (**a,b**) PCA on top 500 DEGs for HUVEC on soft 3 kPa (turquoise circles) and on stiff 70 kPa (purple circles) matrices and for HMEC-1 on soft 3 kPa (orange circles) and on stiff 70 kPa (green circles) matrices. PC1 versus PC2 is shown in panel A and PC3 versus PC4 is shown in panel B. The 95% confidence ellipse is shown for each group with the corresponding colors. (**c**) Scree plot showing in decreasing order the proportion of variance explained by each PCA mode up to PC10.

Principal component analysis of the top 500 most variable genes across all samples confirmed that the EC type but not ECM stiffness is the major determinant of expression differences and accounts for approximately 99% of the variation between transcriptomes (Fig. 4a,c). ECM stiffness appears to be a minor contributor accounting for <1% of the expression variance for HUVEC only, while HMEC-1 on STIFF fall exactly in the same PCA space as on SOFT, appearing indistinguishable (Fig. 4b). Consistent with this analysis, when we performed hierarchical clustering on the *rlog* transformed counts and created dendrograms of the Euclidean distance between pairs of samples, we found that the separation emerges based on cell type but not on subendothelial stiffness (Supplementary Fig. S7a). Similarly, hierarchical clustering on the 200 most variant genes across all samples clearly separates the two EC types, while subendothelial stiffness does not separate HMEC-1, but separates HUVEC with low confidence (Supplementary Fig. S7b and Table S6).

Next, given that the differences between the two EC types are so dramatic, we sought to understand what major biological pathways differ, and to that end we performed Kyoto Encyclopedia of Genes and Genomes (KEGG) pathway analysis and Gene Ontology (GO) functional enrichment for the DEGs of HUVEC versus HMEC-1^35^. Pathway enrichment analysis reveals 9 KEGG pathways significantly perturbed between the two EC types with 4 of those being clearly pertinent to endothelial biomechanics (Supplementary Table S7). Cell adhesion molecules and particularly tight junction genes essential for proper intercellular stress transmission and EC barrier integrity are upregulated for HUVEC as compared to HMEC-1 (*hsa04514*; Supplementary Fig. S8). Most genes related to arachidonic acid metabolism, important for the regulation of vascular tone in response to shear stresses, are also upregulated for HUVEC^36^ (*hsa00590;* Supplementary Fig. S8). Additionally, genes related to ECM-receptor interactions are significantly different between the two EC types with HMEC-1 expressing significantly more collagen (and exclusively COL1A1), tenascin and vitronectin as compared to HUVEC which express more laminin, perlecan and fibronectin among other ECM proteins (*hsa04512;* Supplementary Fig. S9). Finally, genes related to calcium signaling, involved in shear stress- and matrix stiffness-sensing of ECs^37^, are upregulated for HUVEC as compared to HMEC-1 (*hsa04020;* Supplementary Fig. S9). Among the other genes upregulated by HUVEC is myosin light chain kinase (MLCK) central in cellular contractile behavior (Supplementary Fig. S9). Altogether these results suggest that HUVEC as compared to HMEC-1 might be more sensitive and responsive to mechanical cues and perturbations.

To confirm the KEGG results we also mapped the DEGs between HUVEC and HMEC-1 to GO Biological Process (BP) terms. The resulting functional analysis revealed that the majority of significantly perturbed GO terms in the BP category are upregulated for HUVEC as opposed to HMEC-1 (Supplementary Table S8). Interestingly, all the 11 GO terms that are upregulated for HMEC-1 correspond to cell cycle or cell division. In contrast, the 47 upregulated terms found for HUVEC are largely related to endothelial-specific functions, many of which are central in EC biomechanical behavior, in accordance with the KEGG pathways analysis (*e.g*. EC migration, calcium-dependent cell-cell adhesion).

### TGF-β2 and TGF-β2-related genes are among the stiffness-sensitive DEGs identified in HUVEC

When we looked more closely on the 24 stiffness-sensitive DEGs in HUVEC, we found that 8 out of the 24 DEGs correspond to pseudogenes, uncharacterized genes, mitochondrial RNA or microRNA and were therefore not examined further (Supplementary Table S1). Out of the remaining 16 genes, 14 of them are upregulated on stiff and only 2 on soft matrices. Of the 14 genes that are upregulated on stiff matrices, 5 genes encode proteins that are secreted to or remodel the ECM of ECs: TGF-β2, ADAMTSL1, LAMB3, SMOC1, STC1^38,39^. TGF-β2 is a cytokine that is secreted to the ECM in a latent form. Once it switches into an active conformation, TGF-β2 can bind to TGFβ receptors and modulate the synthesis and accumulation of ECM components and the expression of cell surface receptors for ECM components^40–42^. ADAMTSL1 is a latent TGF-β binding protein that is key in ECM remodeling^43^, LAMB3 encodes the β3 subunit of laminin which is a protein of EC basement membranes and is expressed more in response to TGF-β2^44^, SMOC1 is a matricellular protein (*i.e*. does not have a structural but rather a regulatory role when secreted to the ECM) found in basement membranes and is also a TGF-β regulator^39^ and STC1 is a secreted glycoprotein found downstream of TGF-β2 in osteoclasts^38^. From the 11 remaining upregulated genes 4 affect gene expression or transcription, namely: ZNF703, a transcriptional co-repressor related to TGF-β^45^; ZSCAN31, a DNA-binding transcription factor; ID1, a transcriptional regulator^46^; and KLF10, a zinc finger DNA-binding protein that regulates gene expression and is also known as transforming growth factor-β (TGFβ) inducible early gene-1 (*TIEG1*)^47^. In addition, we found the following genes to be upregulated: CXCL12, a secreted chemokine that contributes to cancer^48^; GADD45B an effector of TGF-β signaling^49,50^; KRT7 a cytoskeletal protein expressed in blood vessels and upregulated in response to TGF-β^51^; INSR a transmembrane receptor that is activated by insulin^52^; and EPHA5 a receptor belonging to the protein-tyrosine kinase family^53^. Finally, the only two upregulated genes discovered for HUVEC grown on soft matrices were XAF1 an antagonist of XIAP (suppresses caspase-3 activation and cell death) activities and is suppressed in response to TGF- β stimulation^54^ and OFML3 a secreted scaffold protein important in endothelial to mesenchymal transition (EndMT)^55^.

DE analysis of HMEC-1 monolayers residing on soft versus stiff gels led to the identification of only 7 DEGs (Supplementary Table S2). 6 of them were upregulated on stiff gels and consisted of histone-related genes, along with a pseudogene and a mitochondrially-encoded dehydrogenase. The one gene that was upregulated on soft matrices corresponded to SLC6A6, which encodes a multi-pass membrane protein, a member of a family of sodium and chloride-ion dependent transporters^56^. Overall, HUVEC express more ECM stiffness-sensitive genes as compared to HMEC-1, and seem to retain more EC specific functions and mechanosensitivity according to the pathway enrichment analysis. Given these findings, we decided to focus our attention on this specific cell type to further explore if and how the stiffness-sensitive DEGs we identified are related to each other and to HUVEC mechanobiology.

### RT-qPCR on HUVEC treated with recombinant TGF-β2 confirms changes in expression of several genes identified to be stiffness-sensitive through RNA sequencing

One of the ECM stiffness-sensitive genes that we identified to be upregulated in HUVEC on stiff as opposed to soft gels is TGF-β2. Interestingly, for certain cell types there is a correlation between expression of TGF-β2 and enhanced cellular contractility^57,58^ together with deposition of ECM components^59^ that might even contribute to the development of fibrotic diseases^60^. Triggered by our finding and these past studies, we hypothesized that the differential regulation of many of the genes expressed by HUVEC on stiff but not on soft matrices could be primarily due to TGF-β2 upregulation. To assess the plausibility of this hypothesis, we seeded cells on soft or stiff matrices in monolayers and treated them for 24 h with either vehicle control, or 1 ng/mL or 10 ng/mL of recombinant TGF-β2. We then performed RT-PCR to assess which genes are differentially expressed in the presence or absence of TGF-β2 as compared to controls and whether that depends on subendothelial stiffness (Supplementary Table S9).

**Figure 5:**
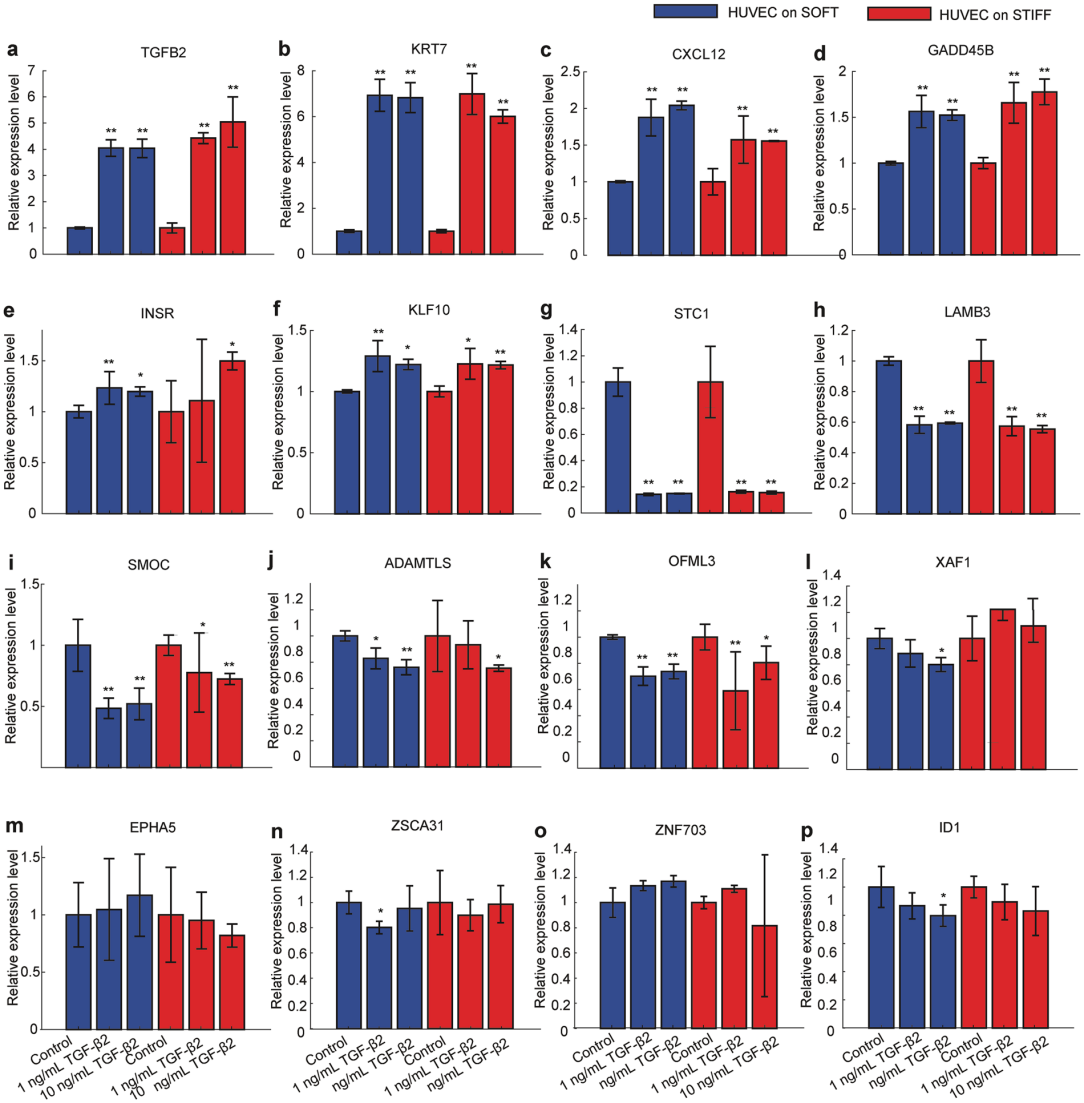
TGF-β2 modulates expression of some stiffness-sensitive genes in HUVEC. (**a–p**) Relative with respect to GAPDH expression levels of the indicated stiffness-sensitive DEGs obtained by RT-qPCR. For each boxplot N = 3 replicates are shown for each group treated with either vehicle control (#1, #4), or treated for 24 h with 1 ng/mL (#2, #5) or 10 ng/mL TGF-β2 (#3, #6). HUVEC either were residing on soft 3 kPa (blue) or stiff 70 kPa (red) matrices and the relative levels of expression in each treated sample (#2-#4 or #6-#8) are expressed relative to the vehicle control samples of cells residing on soft (#1) or stiff (#4) matrices respectively.

We found that addition for 24 h of recombinant TGF-β2 onto HUVEC residing on soft matrices led to a 4-fold or to a 5-fold increase in endogenous TGF-β2 expression for cells residing on soft or stiff matrices respectively (Fig. 5a). Consistent with the idea that TGF-β2 is upstream of several of the DEGs identified, we additionally found a 7-fold increase in expression of KRT7, 2-fold increase of CXCL12, 1.5-fold increase of GADD45B, 1.3-fold increase of INSR and 1.3-fold increase of KFL10 (Fig. 5b–f). Also consistent with the DE analysis, addition of TGF-β2 led to 30% decrease in OFML3 and in 20% decrease of XAF1 for cells residing on stiff matrices only (Fig. 5k,l). To our surprise STC1, LAMB3, SMOC and ADAMTLS (the DEGs expressing proteins involved in ECM remodeling) showed decreased expression compared to cells treated with vehicle control irrespective of substrate stiffness (Fig. 5g–j). STC1 showed 80% decrease in expression, LAMB3 40% decrease, SMOC1 50% decrease and ADAMTLS a 20% decrease (Fig. 5g–j) as compared to control cells. EPHA5, ZSCAN31, ZNF703 and ID1 did not show any appreciable changes in gene expression irrespective of the treatments (Fig. 5m–p). These results suggest that at least 7 of the DEGs identified when comparing HUVEC on stiff versus soft matrices are regulated downstream of TGF-β2.

### HUVEC but not HMEC-1 treated with recombinant TGF-β2 show a significant elevation in their traction stresses on both stiff and soft matrices

**Figure 6:**
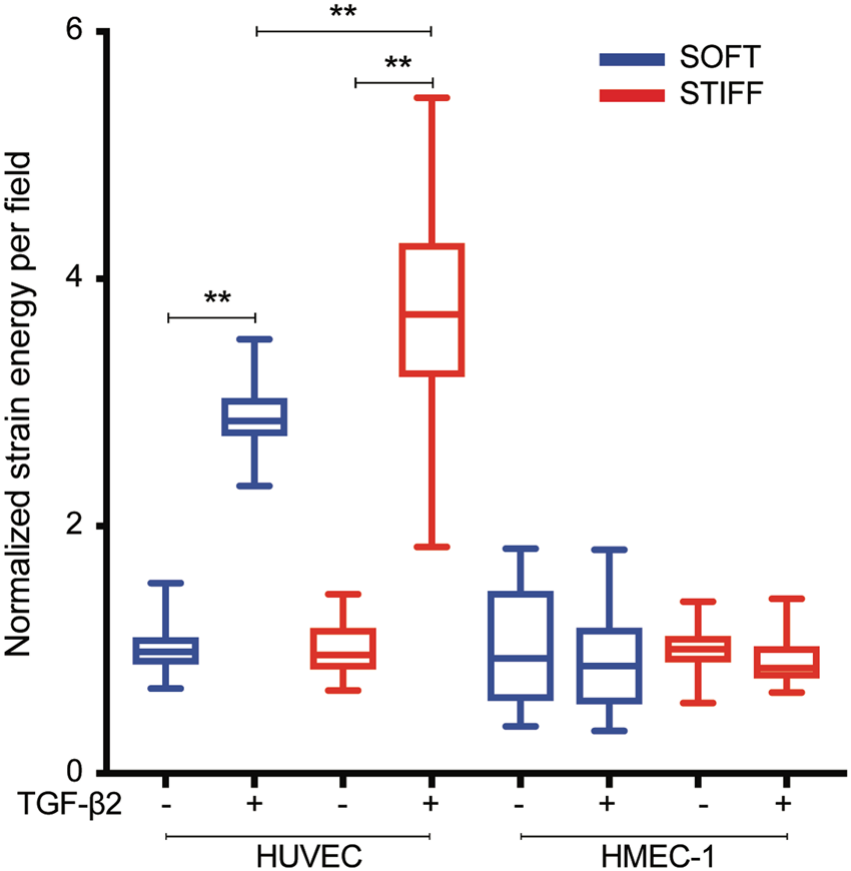
HUVEC but not HMEC-1 treated with recombinant TGF-β2 show a significant elevation in their traction stresses on both stiff and soft matrices. Boxplots of the strain energy imparted by confluent EC monolayers calculated during different instants of time and for multiple fields of view (N = 200). Boxplots refer to HUVEC or HMEC-1 residing on soft 3 kPa (blue) or stiff 35 kPa matrices (red) treated with vehicle control or 1 ng/mL TGF-β2 for 24 h prior to imaging. Each boxplot is normalized with respect to the mean value of the vehicle control case. Two asterisks denote statistically significant differences between the medians of two distributions (p < 0.01; Wilcoxon rank sum test).

We next asked whether TGF-β2 and its downstream effectors might contribute to the elevation in the traction stresses that HUVEC display on stiff as opposed to soft matrices. For that we grew HUVEC in monolayers on soft 3 kPa or stiff 35 kPa hydrogels and added 1 ng/mL TGF-β2 or vehicle control for 24 h. For comparison, we also examined if addition of recombinant TGF-β2 would influence HMEC-1 mechanics by performing in parallel TFM on this cell type. Consistent with our previous findings, we found that both HMEC-1 and HUVEC when treated with vehicle control exert higher traction stresses on stiffer as opposed to softer matrices (Supplementary Figs. S10 and S11). When HUVEC were treated with TGF-β2 they displayed an elevation of their cell-matrix deformations, traction stresses and strain energy as compared to controls both on soft and stiff matrices (Supplementary Fig. S10a–c; Supplementary Movies S1 and S2). Addition of TGF-β2 led to a higher increase in strain energy for HUVEC residing on stiff (4-fold increase) as opposed to soft (3-fold increase) matrices (Fig. 6). We also found that cells are less dynamic upon addition of TGF-β2 since the correlation of the deformation maps between subsequent frames decreased much faster for cells treated with vehicle control as compared to cells treated with TGF-β2 (Supplementary Fig. S12). Unlike HUVEC, HMEC-1 did not increase either their traction stresses or strain energy upon addition of TGF-β2 (Fig. 6 and Supplementary Fig. S10a–c). Indeed, when we plotted the mean strain energy per field of view for multiple recordings and normalized it for each condition (cell type or stiffness) with the strain energy of vehicle control, we found an approximately 2 or 3-fold increase in the strain energy for HUVEC residing on soft or stiff matrices respectively but no change for HMEC-1 irrespective of stiffness (Fig. 6).

### HUVEC exposed to TGF-β2 show increased amount of F-actin and pMLC

**Figure 7:**
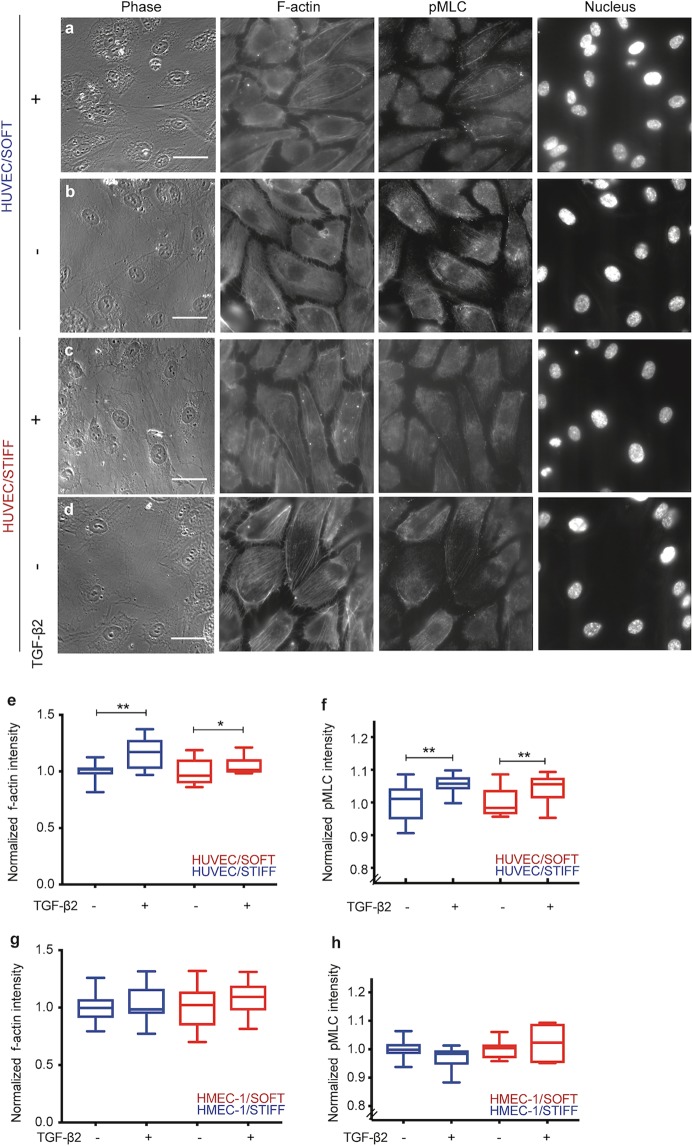
F-actin and pMLC increase for HUVEC monolayers exposed to TGF-β2. (**a–d**) Representative images depicting the phase image of cells (first column), F-actin fluorescence (second column), anti-pMLC antibody fluorescence (third column) and the image of the nuclei (fourth column) for HUVEC residing on soft 3 kPa matrices and treated with vehicle control (**a**) or 1 ng/mL TGF-β2 for 24 h (**b**) or HUVEC residing on stiff 70 kPa matrices and treated with vehicle control (**c**) or 1 ng/mL TGF-β2 for 24 h (**d**). Scale bar is 50 μm. (**e,f)** Boxplots showing the integral of the F-actin fluorescence intensity (**e**) or pMLC (**f**) over different fields of view (N = 10) for HUVEC residing on soft 3 kPa matrices (blue) and treated with vehicle control or 1 ng/mL TGF-β2 for 24 h and for HUVEC residing on stiff (red) 70 kPa matrices and treated with vehicle control or 1 ng/mL TGF-β2 for 24 h. N = 400–500 cells were analyzed for each condition. One or two asterisks denote statistically significant differences between the medians of two distributions (<0.05 or <0.01 respectively; Wilcoxon rank sum test). (**g,h)** Same as panels e-f but for HMEC-1 cells.

The increase in cell-matrix traction stresses that HUVEC exert upon addition of TGF-β2 suggest major actin cytoskeletal rearrangements occurring within the cells or changes in their actomyosin contractility or both. To explore this possibility, we seeded HUVEC on soft (3 kPa) and stiff (70 kPa) hydrogels and added recombinant TGF-β2 or vehicle control for 24 h. Samples were then fixed and stained with phalloidin to visualize F-actin and DAPI to stain the cells’ nuclei, to ensure that confluency is similar between different conditions (Supplementary Fig. S13a,b). Since actomyosin contractility is mediated through the phosphorylation of myosin regulatory light chain (MLC)^61^, we also used an antibody against pMLC (Thr18/Ser19) to assess potential changes in myosin activity that could explain the increase in traction stresses upon addition of TGF-β2. As shown by the representative images in Fig. 7, HUVEC on stiff as opposed to soft gels showed slightly increased F-actin fluorescence intensity and increased actin stress fiber formation, reinforcing the idea that actin organization rather than gene expression is what different in these two distinct mechanical regimes (Fig. 7a,c). In addition, samples of wells treated with TGF-β2 showed a significant increase both in F-actin fluorescence intensity and in actin stress fiber formation, particularly on soft but also on stiff substrates (Fig. 7b,d,e). In parallel, we investigated the effect of TGF-β2 on the phosphorylation of MLC and found an increase in pMLC fluorescence intensity for HUVEC treated with TGF-β2 residing on both soft and stiff substrates, similar to the increase in F-actin (Fig. 7b,d,f). In addition, pMLC appeared in part to co-localize with F-actin stress fibers, suggesting that F-actin reorganization and actomyosin contractility in response to TGF-β2 for both substrate stiffnesses might in part explain the increase in traction stresses for HUVEC. We also immunostained HMEC-1 under the same conditions but were unable to see any changes in F-actin or pMLC intensity and localization, upon addition of TGF-β2, consistent with the absence of changes in the magnitude of traction stresses these cells exert upon addition of TGF-β2 (Fig. 7g,h).

## Discussion

In this study we showed for the first time that subendothelial stiffness has a minimal effect on the transcriptome of HUVEC and HMEC-1 cells when those are seeded in confluence for 24 h on collagen I-coated hydrogels. The effect of ECM stiffness on the transcriptome of cells has been examined before for certain cell types and has yielded novel insight into the importance of stiffness in dictating the cellular transcriptome^12–14^. In hepatocytes 4000 genes were differentially-regulated depending on ECM stiffness although cell confluency in this study was not explicitly stated^14^. Similarly, the mesenchymal stem cell transcriptome was found extremely stiffness-sensitive when these cells are sparsely encapsulated within alginate gels^12^. When vascular smooth muscle cells were placed on soft or stiff matrices, 2842 stiffness-sensitive genes were identified common in both cell types corresponding to both protein coding and lncRNAs^13^, although no fold change cutoff was considered for DEG identification in this study. Interestingly, in most of these studies the characterized cells were not plated under conditions where they formed monolayers or they were plated as single cells. Single or subconfluent ECs placed on matrices of varying stiffness show much more intense differences in their morphology, cytoskeletal architecture and mechanics as compared to cells placed in confluency and studies have shown that cell confluency can even override the effect of matrix stiffness^5,20,62^. In this study we chose to examine ECs in confluency, as this scenario is more physiologically relevant (*i.e*. ECs are not found *in vivo* as single cells, but in a healthy endothelium cells are confluent and non-proliferating).

We discovered that both confluent HUVEC and HMEC-1 exert higher traction forces when residing on stiff as compared to soft substrates but this change in mechanical behavior does not originate from differential regulation of gene expression. It is likely that post-transcriptional regulation might control ECM stiffness-sensitive responses of ECs^63^. In accordance with this argument and past studies, we found that the phosphorylation level or the conformation of key mechanosensitive proteins is differentially regulated depending on subendothelial stiffness^28–30^. It could also be that the changes in traction forces arise due to changes not in the amount of genes expressed but rather on the localization of the corresponding proteins. In the past we discovered increased amounts of surface vimentin for HMEC-1 on stiffer matrices, while the total amount of vimentin remained the same between conditions^32^. Similarly, herein we found increased actin stress fiber formation for HUVEC on stiff versus soft matrices although no changes in the expression of the ACTB gene were detected (Fig. 6a,c). Finally, miRNAs, that typically do not affect gene expression but rather the translation and stability of mRNAs^64,65^, rather than mRNA or lncRNA can play a critical role in EC mechanosensing and mechanotransduction^16,17^. These mechanosensitive miRNAs can even contribute in arterial stiffening, fibrosis and hypertension^16,66^, however we did not assess miRNA expression in this study.

An important conclusion of our study is that, unlike primary HUVEC that exhibit 24 stiffness-sensitive DEGs when grown on stiff versus soft matrices, HMEC-1 cells express fewer DEGs, appearing stiffness-insensitive in a transcriptomic level. However, when comparing HUVEC to HMEC-1 irrespective of substrate stiffness gene expression differences are dramatic (Fig. 2). Through functional analysis of DEGs, we found that multiple pathways related to proliferation and cell cycle regulation are upregulated for HMEC-1, whereas HUVEC show upregulation of many more pathways related to endothelial functions^67^. This behavior of HMEC-1 might arise from their immortalized nature since immortalized human EC lines demonstrate significant differences in their ability to respond to cytokines compared to primary ECs^21,68^. Whether the differences of primary HUVEC versus HMEC-1 are caused by immortalization procedures of the latter or just reflect heterogeneity of ECs arising from different vascular beds could be the focus of future studies.

From the 24 stiffness-sensitive DEGs identified for HUVEC, we decided to focus our attention on TGF-β2 cytokine for several reasons: (1) It has been shown to be mechanosensitive. It is secreted in a latent form on the ECM and can switch into an active conformation by mechanical force applied by the cells onto their matrix on which it is embedded. That allows active TGF-β2 to bind to TGF-β receptors eliciting a cascade of events crucial for EC homeostasis^40–42^ but which also when unregulated can contribute to the development of fibrosis, cardiovascular disease, or may be usurped during tumor growth^59^; (2) TGF-β2 is also implicated in ECM protein production that is thought to lead to tissue remodeling and fibrosis characterized by ECM stiffening^69^. Indeed a lot of the DEGs we identified to be upregulated on stiff matrices are ECM proteins^38,39^; (3) Previous literature suggests direct or indirect relationship with TGF-β2 for most of the identified DEGs^38,44–52,54,55^; (4) Recently TGF-β2 was shown to be downregulated in lymphatic ECs seeded on soft as opposed to stiff matrices^70^.

Indeed, we showed that addition of recombinant TGF-β2 increases endogenous TGF-β2 more for cells residing on stiff and to a lesser degree for cells residing on soft matrices. Together with the observation that traction stresses for HUVEC on stiff matrices are higher than on soft, this result might be suggestive of the cells being able to mechanically activate more easily the latent TGF-β2 on stiff matrices which then leads to more endogenous TGF-β2 expression compared to soft matrices. We also discovered that upon addition of recombinant TGF-β2 most of the DEGs identified to be up- or down- regulated on stiff as compared to soft matrices followed similar trends, suggestive of these genes being downstream of TGF-β2. The only genes that followed the opposite trend were STC1, LAMB3, SMOC and ADAMTLS which showed decreased expression compared to cells treated with vehicle control irrespective of substrate stiffness. We speculate that this result might be due to the high potentially non-physiological amount of added TGF-β2 or to the fact that these genes are just not regulated via TGF-β2. For instance, increased STC1 levels have shown to decrease TGF-β2 expression and reduce fibrotic effects resulting from it^71^.

We hypothesized that if the increase in traction stresses that HUVEC exert on stiff matrices is partially due to enhanced TGF-β2 expression, then addition of recombinant TGF-β2 that enhances endogenous TGF-β2 expression should lead to an increase in the traction stresses that HUVEC exert. Indeed, upon addition of recombinant TGF-β2 we found a 2-fold and 3-fold increase in the strain energy that HUVEC exert on soft and on stiff matrices respectively. Activation of extracellular TGF-β from its latent state to an active state can happen via cells mechanical pulling the latent complex via their integrin based focal adhesions^59^. Matrix elasticity might also play a role in TGF-β activation, as shown for myofibroblasts^72^. However, it is not conclusive whether increased matrix stiffness allows cells to grab stronger thereby mechanically activating TGF-β2 and leading to subsequent enhancement in production of TGF-β2. Our findings are suggestive of such a mechanism because addition of recombinant TGF-β2 leads to further increase in endogenous TGF-β2 for cells residing on stiff as opposed to soft matrices. Consistent with this idea, when we measure the traction force increase that HUVEC produce on their matrix upon addition of recombinant TGF-β2 we measure a 3-fold increase in strain energy, as opposed to a 2-fold increase on softer matrices.

TGF-β2 has been previously implicated in modulating cellular contractility for certain cell types^73,74^. In these studies, active TGF-β2 by binding to TGF-β receptors elicits a cascade of downstream signaling either through the canonical Smad-dependent pathway or through non-canonical pathways in which MAPK and ROCK/RhoA are involved inducing increased actin polymerization and/or actomyosin contractility^58,74^. Our immunofluorescence imaging of HUVEC treated with recombinant TGF-β2 are consistent with the idea that activation of TGF-β2 in HUVEC, signals most probably through the non-canonical RhoA/ROCK pathway leading to increased actomyosin contractility and elevated cell-ECM traction stresses. In turn that appears to lead to further activation of TGF-β2, synthesis of additional ECM molecules and expression of more TGF-β2. Unlike HUVEC, HMEC-1 do not respond mechanically to addition of TGF-β2, a discrepancy which is consistent with TGF-β2 not being a stiffness-sensitive DEG for HMEC-1. Different EC types are well known to exhibit local morphological and functional specializations and distinct gene expression profiles depending on the tissue they originate from^75^ and can therefore respond differently to cytokines including TGF-β2^76^. Which EC types are sensitive to TGF-β2 and how much that depends on the anatomical location they come from or their local microenvironment, including its mechanics, could be the focus of future studies.

## Materials and Methods

### Fabrication of polyacrylamide hydrogels on multi-well plates

Polyacrylamide hydrogel fabrication was done as previously described^77^. Glass-bottom plates with 24 wells (MatTek; P24G-1.5-13-F) were incubated for 1 h with 500 μL of 1 M NaOH, then rinsed with distilled water, and incubated with 500 μL of 2% 3-aminopropyltriethoxysilane (Sigma; 919-30-2) in 95% ethanol for 5 min. Following rinsing with water 500 μL of 0.5% glutaraldehyde were added to each well for 30 min. Wells were rinsed with water again and dried at 60 °C. To prepare polyacrylamide hydrogels of varying stiffness, mixtures containing 3–10% acrylamide (Sigma; A4058) and 0.06–0.6% bis-acrylamide (Fisher; BP1404–250) were prepared. Specifically, 3 kPa hydrogels contained 5% acrylamide and 0.1% bis-acrylamide, 35 kPa hydrogels contained 8% acrylamide and 0.26% bis-acrylamide, and 70-kPa hydrogels contained 10% acrylamide and 0.6% bis-acrylamide^77^. For all experiments described herein we chose 3 kPa stiffness for our “SOFT” ECM condition, since it is the lowest stiffness where we can still attain confluent monolayers. Below 3 kPa neither cell type (HUVEC or HMEC-1) consistently forms monolayers and cells often cluster or form vessel like structures with gaps as previously shown^26,27^. For all experiments other than TFM, we used 70 kPa for our “STIFF” ECM condition given that this stiffness lies on the upper range of what can be encountered *in vivo*^22–24^. However, when we performed TFM on cells at 70 kPa the gels were too stiff to enable cells to deform them so that we could consistently capture displacements of the beads (embedded into the hydrogels on which cells are seeded). We therefore used only for TFM 35 kPa hydrogels as “STIFF” matrices since this is the highest stiffness on which cells are still able to consistently deform the hydrogels. For each stiffness, two mixtures were prepared, the second of which contained 0.03% 0.1 μm–diameter fluorescent beads (Invitrogen, F8803) for TFM experiments.

0.06% APS and 0.43% TEMED were added to the solutions to initiate polymerization. First, 3.6 µL of the first mixture without the beads was added at the center of each well, capped with 12-mm untreated circular glass coverslips, and allowed to polymerize for 20 min. Then 2.4 µL of the mixture containing tracer beads was added, sandwiched again with a 12-mm untreated circular glass coverslip and allowed to polymerize for 20 min. Next, 50 mM HEPES at pH 7.5 was added to the wells, and coverslips were removed. Hydrogels were UV-sterilized for 1 h and then activated by adding 200 µL of 0.5% weight/volume heterobifunctional cross-linker Sulfo-SANPAH (ProteoChem; c1111) in 1% dimethyl sulfoxide (DMSO) and 50 mM HEPES, pH 7.5, on the upper surface of the hydrogels and exposing them to UV light for 10 min. Hydrogels were washed with 50 mM HEPES at pH 7.5 and were coated with 200 µL of 0.25 mg/ml rat tail collagen I (Sigma-Aldrich; C3867) in 50 mM HEPES at pH 7.5 overnight at room temperature. Note that for all assays other than TFM, stiff gels were 70 kPa. For TFM only stiff gels were 35 kPa because that was determined to be empirically the limit of the ECs being able to still deform their matrix. On 70 kPa hydrogels we were unable to detect any matrix deformations (data not shown).

### Atomic force microscopy (AFM) for determination of hydrogel stiffness

AFM force-distance measurements were performed on polyacrylamide hydrogel samples immersed in 50 mM HEPES pH 7.5 buffer with a Park NX-10 AFM (Park Systems, Santa Clara, CA) using silicon nitride cantilevers CP-PNP-SiO with a sphere tip (sQube, 0.08 N/m stiffness, sphere radius ~1 μm) and gold coating on the reflective side. Temperature was 37 °C throughout the experiment. Tip calibration curves were performed on a glass surface considered to be infinitely hard for the soft tips used and two approach-withdraw cycles were performed. The XEI (Park Systems, Santa Clara, CA) and SPIP softwares (Image Metrology, Hørsholm, Denmark) were used for data analysis of the FD curves and for calculation of the gel stiffness.

### Cell culture conditions

HMEC-1 (generous gift from the Welch lab, University of California, Berkeley) were cultured in MCDB 131 medium (Fisher Scientific; 10372–019) supplemented with 10% FBS (GemBio; 900-108), 10 ng/mL epidermal growth factor (Sigma; E9644), 1 μg/mL hydrocortisone (Sigma; H0888), and 2 mM l-glutamine (Sigma; 56-85-9). HUVEC (Lonza C2517A) were cultured according to the manufacturer’s instructions (EGM Bullet Kit-2, Lonza CC-3162). Passages used were between P4–P8.

### Traction force microscopy

TFM assays were performed as previously described^25,78,79^. Two layered polyacrylamide hydrogels were manufactured as described above. After hydrogel equilibration with cell media for 30 min at 37 °C, cells were seeded to a concentration of 2 × 10^5^ cells per well directly onto the hydrogels 24 h prior to imaging. Multi-channel time-lapse sequences of fluorescence (to image the beads within the upper portion of the hydrogels) and phase contrast images (to image the cells) were acquired using an inverted Nikon Diaphot 200 with a CCD camera (Andor Technologies) using a 40X Plan Fluor NA 0.60 objective and the MicroManager software package (Open Imaging). The microscope was surrounded by a cage incubator (Haison) maintained at 37 °C and 5% CO_2_. Images were acquired every 10 min for approximately 8 h. Subsequently, at each time interval we measured the 2D deformation of the substrate at each point using an image correlation technique similar to particle image velocimetry. We calculated the local deformation vector by performing image correlation between each image and an undeformed reference image which we acquired by adding 10% SDS at the end of each recording to detach the cells from the hydrogels. We used interrogation windows of 32 × 8 pixels (window size x window overlap). We calculated the two-dimensional traction stresses that cell monolayers exert to the hydrogel as described elsewhere^78,80^. We calculated the strain energy (*U*_*s*_) as the mechanical work imparted by the cell to deform its hydrogel: ${U}_{s}=\frac{1}{2}{\int }_{s}\overrightarrow{\tau }(z=h)\cdot \overrightarrow{u}(z=h)ds$, where $\overrightarrow{u}$ is the measured displacement vector field on the free surface of the hydrogel and ${\int }_{s}ds$ represents a surface integral.

### Antibodies and reagents

Hoechst (Thermofisher; D1306) was dissolved at 1 mg/ml in DMSO and used at 1:1000 to stain nuclei. Recombinant TGF-β2 (Sigma; T2815) was dissolved in water containing 0.1% BSA at stock concentration 50 μg/mL, stored at −80 °C and was added to cells for 24 h at either 1 ng/mL or 10 ng/mL. Primary antibody used for staining of pMLC (Cell Signaling; 3674S) was rabbit polyclonal phospho-Myosin Light Chain 2 (Thr18/Ser19) antibody. For actin staining 0.2 µM AlexaFluor488 phalloidin (Thermo Fisher; A12379) was used. For western blot analysis, active form of integrin β1 (Millipore; MAB2079), integrin β1 (Millipore; MAB2053), phospho-Tyr-397 FAK (Invitrogen; PA5-17084), FAK (Santa Cruz; sc158), phospho-Vav2 (Santa Cruz; sc16409R), Vav2 (Abcam; AB86699), phospho-ERK (Cell Signaling; 9109S), ERK2 (Santa Cruz; sc154-G), phospho-p70S6K (Cell Signaling; 9205S), p70S6K (Cell Signaling; 9202) and GAPDH (Santa Cruz; sc20538) were used.

### Western blotting for HUVEC or HMEC-1 lysates coming from cells in monolayers residing on different ECM stiffness substrates

To assess phosphorylation and expression levels of different mechanosensitive proteins^28^, cells were seeded at a concentration of 2 × 10^5^ cells/well (24-well plates) on soft 3 kPa or stiff 70 kPa hydrogels for 24 h, and then lysed with a buffer containing 1% Nonidet P-40, 0.5% sodium deoxycholate, and a protease inhibitor mixture (phenylmethylsulfonyl fluoride [PMSF], leupeptin, aprotinin, and sodium orthovanadate). The total cell lysate was separated by SDS–PAGE (10% running, 4% stacking) and transferred onto a nitrocellulose membrane (Immobilon P, 0.45-μm pore size). The membrane was then incubated with the designated antibodies. Immunodetection was performed using the Western-Light chemiluminescent detection system (Applied Biosystems).

### RNA isolation and RNA sequencing

#### Sample preparation

4^th^ passage HUVEC and HMEC-1 cells were placed at a concentration of 2 × 10^5^ cells/well on soft 3 kPa hydrogels or stiff 70 kPa hydrogels (N = 6 replicates for each condition) built on wells of a 24-multi well plate. Cells residing for 24 h on these gels were then harvested and lyzed using the QIAshredder Kit (Qiagen; 79656). mRNA was harvested using the RNeasy Plus Micro Kit (Qiagen; 74004) and eluted in 30 μL RNAase free water. RNA concentrations were measured using the nanodrop machine and were comparable between conditions. RNA quality and quantity were confirmed via bioanalyzer analysis performed by the Stanford Protein and Nucleic Acid (PAN) Facility.

#### Library preparation and RNA sequencing

RNA libraries were prepared by the Stanford Functional Genomics Facility using the KAPA stranded RNA-seq kit with RiboErase (Kit code KK8483, Roche Cat. # 07962282001), for a fragment length of 200–300 bp. Sequencing was run on the Illumina NextSeq. 500 System using the High-Output Kit with 2 × 75 read length. We had an average of 30 million reads per sample. We used 2 lanes with 12 samples each ensuring that from each condition 3 replicates go to lane A and the other 3 to lane B (total number of replicates N = 6).

### Transcriptome assembly and differentially expressed gene identification

Sequencing reads were obtained in Fastq format and evaluated using FastQC v0.11.5 according to the directions on the following website: http://www.bioinformatics.babraham.ac.uk/projects/fastqc/. For sequence alignment we used HISAT2 (https://ccb.jhu.edu/software/hisat2/index.shtml) and reads were mapped to reference genome grch38 to generate.bam files. Python script HTSEQ was then used to generate counts per read (https://htseq.readthedocs.io/en/release_0.11.1/count.html). Both HISAT2 and HTSEQ were run from the command line. For differential gene expression analysis, we used Deseq2R package (Bioconductor version: Release 3.8, https://bioconductor.org/packages/release/bioc/html/DESeq2.html). The thresholds that we used for identifying DEGS were: (1) DESeq. 2 mean normalized counts >10; (2) padj-value < 0.05; and (3) lof2foldchange >0^34^.

### Hierarchical clustering, PCA and gene set analysis

The R package Bioconductor 3.8 and imported the libraries genefilter and RcolorBrewer to perform hierarchical clustering dendrograms based on the Euclidean distance of the sample and on the 200 top variance genes. Principal Component Analysis (PCA) was performed using the plotPCA function in R. Kyoto Encyclopedia of Genes and Genomes (KEGG) pathway and Gene Ontology (GO) term analyses of the whole data set of DEG were performed using the R package GAGE “Generally Acceptable Gene set Enrichment” (GAGE v.2.22.0) package implemented in R. Briefly, default parameter settings were used for comparisons of log-scaled gene set expression (i.e. enrichment) data between different time points (q-value < 0.01). Gene sets were defined using annotations obtained from GAGE v2.22.0, go.db v3.2.2, and kegg.db v3.2.2. The R package “Pathview” v.1.12.0 and KEGGGraph v1.30.0 were used to visualize gene set expression data in the context of functional pathways.

### RT-qPCR

4^th^ passage HUVEC were placed at a concentration of 2 × 10^5^ cells/well on soft 3 kPa hydrogels or stiff 70 kPa polyacrylamide hydrogels (N = 3 replicates for each condition) built on wells of a 24-multi-well plate. Cells residing for 24 h on these gels were treated either with vehicle control or recombinant TGF-β2 for 24 h, without the cells being previously starved. Cells were then harvested and lyzed using the QIAshredder Kit (Qiagen; 79656). mRNA was harvested using the RNeasy Plus Micro Kit (Qiagen; 74004) and eluted in 30 μL RNAase free water. RNA concentrations were measured using the nanodrop machine and were comparable between conditions. cDNA was prepared using the Superscript III First-strand Synthesis SuperMix (Invitrogen; 18080085). RT-qPCR was performed using the SYBR qPCR Master mix by Arraystar Inc. Genes of interest were amplified using primers indicated in Supplementary Table S9. Briefly the steps followed were: (1) Performance of RT-qPCR for each target gene and the housekeeping gene GAPDH; (2) According to the standard curve, the gene concentration of each sample is generated directly by Rotor-Gene Real-Time Analysis Software 6.0; (3) For each sample, the relative amount of the target gene is determined by calculating the ratio between the concentration of the target gene and that of GAPDH.

### Immunostaining

HMEC-1 and HUVEC cells residing on soft 3 kPa hydrogels or stiff 70 kPa polyacrylamide hydrogels were incubated for 24 h with 1 ng/mL TGF-β2 without the cells being previously starved. Prior to fixation 1 μg/mL Hoechst (Thermofisher; D1306) was added in each well to stain the cells’ nuclei for 10 min. Cells were washed once with PBS and fixed with 4% formaldehyde of EM grade in PBS for 10 min. Following a wash with PBS, samples were permeabilized for 5 min in 0.2% Triton X-100 in PBS and then washed again with PBS. Samples were then blocked for 30 min with 5% BSA in PBS and then incubated with anti-pMLC primary antibody (Cell Signaling; 3674S) diluted 1:100 in PBS containing 2% BSA for 1 h. Samples were washed in PBS three times and then incubated with Alexa Fluor 546 goat anti-rabbit IgG secondary antibody (Invitrogen A-11035) diluted 1:250 in PBS containing 2% BSA for 1 h and 0.2 µM AlexaFluor488 phalloidin. Samples were washed three times in PBS and stored in 1 mL PBS for imaging. *N* > 500 cells were analyzed per condition. For imaging, we used an inverted Nikon Diaphot 200 with a charge-coupled device (CCD) camera (Andor Technologies) and a 60× air Plan Fluor NA 0.60 or a 100× oil objective. The microscope was controlled by the MicroManager software package.

## Supplementary information

Supplementary Figures and Captions

Supplementary Movie 1

Supplementary Movie 2

Supplementary Table 1

Supplementary Table 2

Supplementary Table 3

Supplementary Table 4

Supplementary Table 5

Supplementary Table 6

Supplementary Table 7

Supplementary Table 8

Supplementary Table 9

## Data Availability

The RNA sequencing data (FASTq files) generated during this study and subsequent analysis have been submitted to the Gene Expression Omnibus (GEO) database. These data are available at: https://www.ncbi.nlm.nih.gov/geo/query/acc.cgi?acc=GSE135123 and the series record that provides access to all of the data is GSE135123. All the differential expression analysis results of this study are included as supplementary tables in this article.
