# Supplementary material for: Subendothelial stiffness alters endothelial cell traction force generation while exerting a minimal effect on the transcriptome
Source: Sci Rep. 2019 Dec 3;9:18209. doi: 10.1038/s41598-019-54336-2 (PMC6890669; doi:10.1038/s41598-019-54336-2)
Supplement: Supplementary file 1 — Supplementary Figures and Captions [file 41598_2019_54336_MOESM1_ESM.pdf]

SUPPLEMENTARY INFORMATION

Subendothelial stiffness alters endothelial cell traction force generation while exerting a minimal effect on the transcriptome

Effie E. Bastounis[1], Yi-Ting Yeh[2], and Julie A. Theriot[1, +]

[1] Department of Biology and Howard Hughes Medical Institute, University of Washington, Seattle, WA 98195-1800

[2] Department of Bioengineering, University of California San Diego, La Jolla, California, USA

[+] Corresponding Author:

Julie A. Theriot, Ph.D.

Professor, Department of Biology, University of Washington and Howard Hughes Medical Investigator

Department of Biology and Howard Hughes Medical Institute, University of Washington, Box 351800, Seattle, WA 98195-1800

Email: jtheriot@uw.edu, phone: (206) 543-3397

SUPPLEMENTARY FIGURES

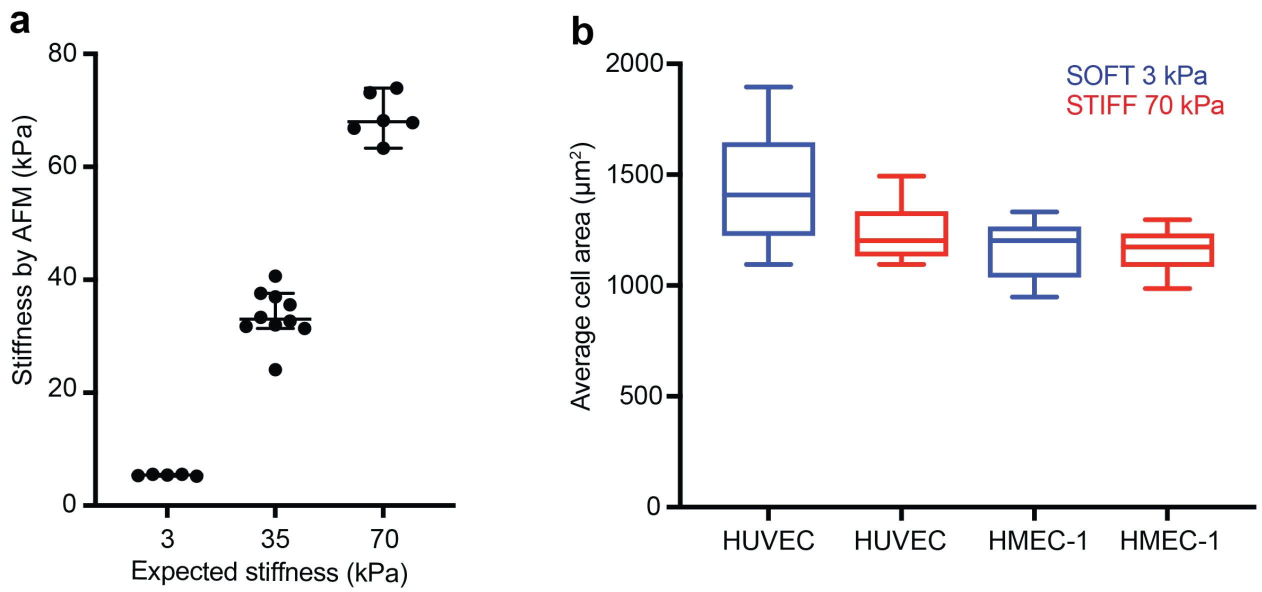

Supplementary Figure S1. AFM measurements of hydrogel stiffness and mean cell area on varying stiffness hydrogels. (**a**) Plot showing the expected Young's modulus of the hydrogels given the amount of acrylamide and bis-acrylamide used (x-axis) versus the Young's modulus measured through AFM (y-axis). Individual circles correspond to individual indented gels, the horizontal bar depicts the data mean and the vertical bars the 95% confidence interval. (**a**) Boxplots of the average cell area across different fields of view (N=9) for HUVEC or HMEC-1 residing on soft 3 kPa hydrogels (red) or stiff (70 kPa hydrogels). Average cell area was calculated by dividing the total area of the field of view by the number of nuclei contained within it. Vertical bar on boxplots represents the data's mean, whiskers the 5-95 percentile and boxes the interquartile range. *P*-values between cell types were calculated with the non-parametric wilcoxon ranksum test and differences were non-significant.

a

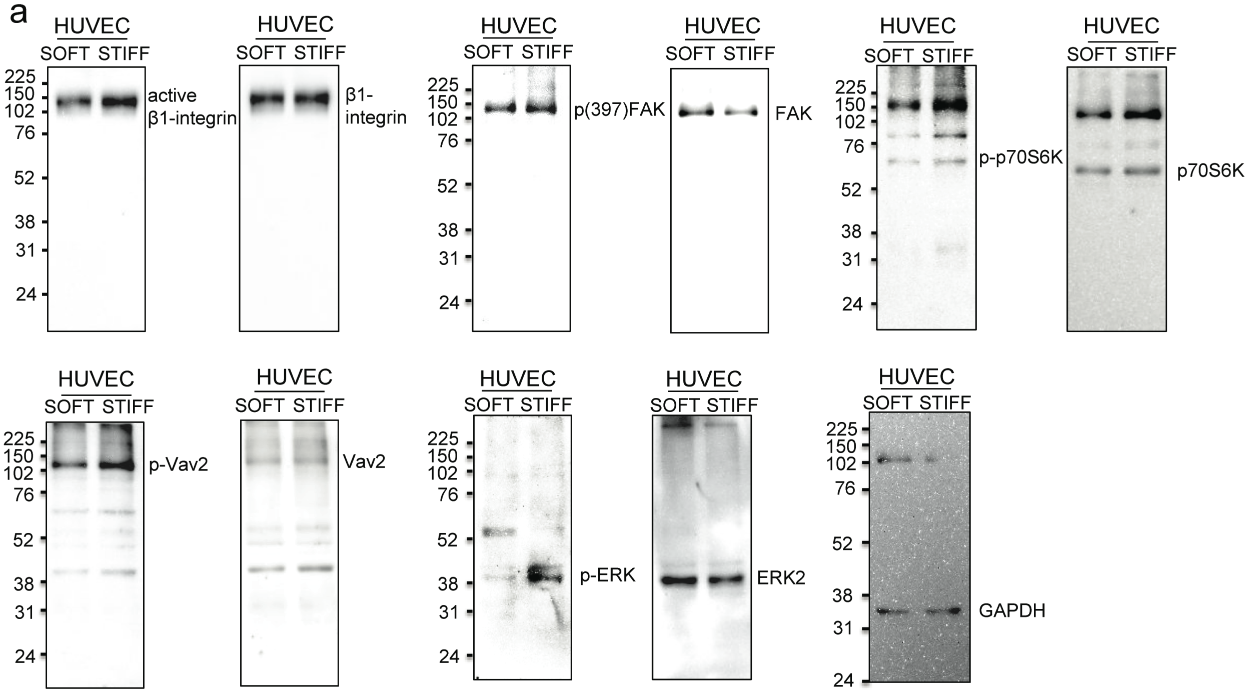
HUVEC
SOFT STIFF
225
150
102
76
52
38
31
24
active β1-integrin
β1-integrin
p(397)FAK
FAK
p-p70S6K
p70S6K
p-Vav2
Vav2
p-ERK
ERK2
GAPDH

b

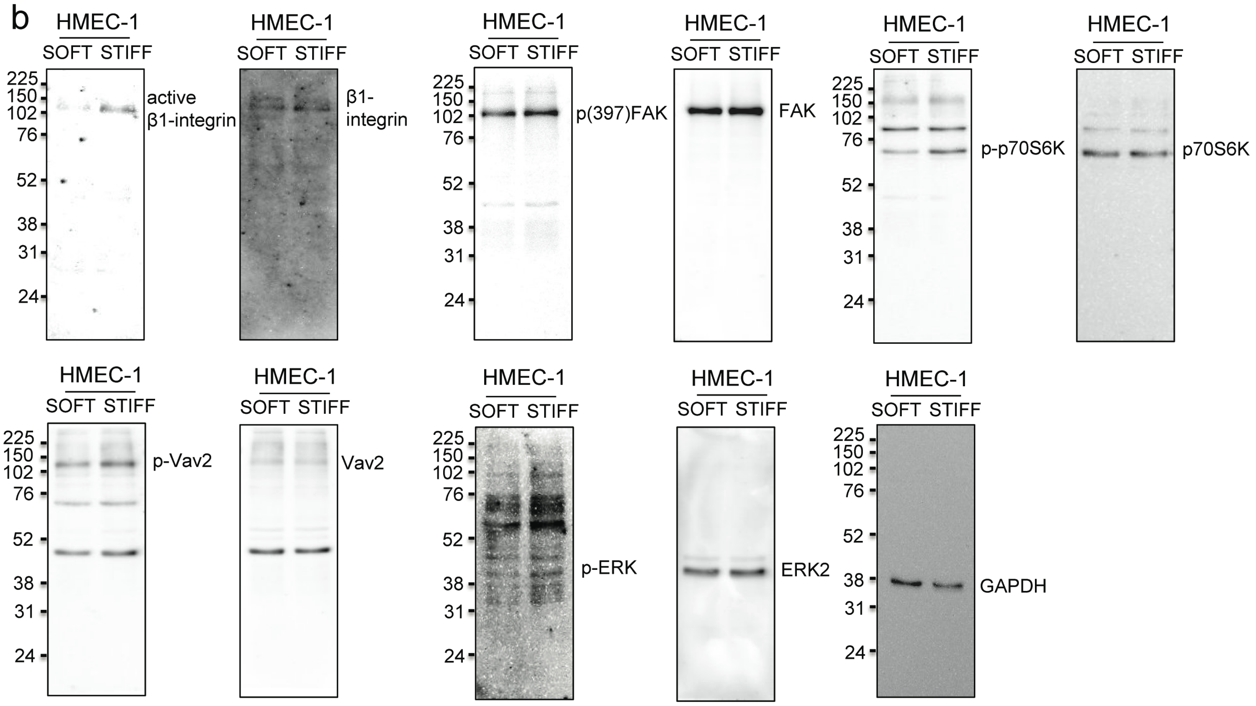
HMEC-1
SOFT STIFF
225
150
102
76
52
38
31
24
active β1-integrin
β1-integrin
p(397)FAK
FAK
p-p70S6K
p70S6K
p-Vav2
Vav2
p-ERK
ERK2
GAPDH

Supplementary Figure S2. Post-transcriptional changes on ECs in monolayers grown on soft versus stiff hydrogels. Supplementary figure refers to Figure 4. Representative cropped blots shown in Fig. 4a are shown here in full display. (**a-b**) Panel (a) refers to HUVEC and panel (b) to HMEC-1 grown on soft (3 kPa) or stiff (70 kPa) substrates. Each blot refers to a different protein whose expression or phosphorylation state was probed namely: active form of integrin β1, integrin β1, p(397)FAK, FAK, p-p70S6K, p70S6K, p-Vav2, Vav2, p-ERK, ERK2 and GAPDH (used as loading control). For details regarding the source of the antibodies used please refer to Material and Methods Section “Antibodies and Reagents”.

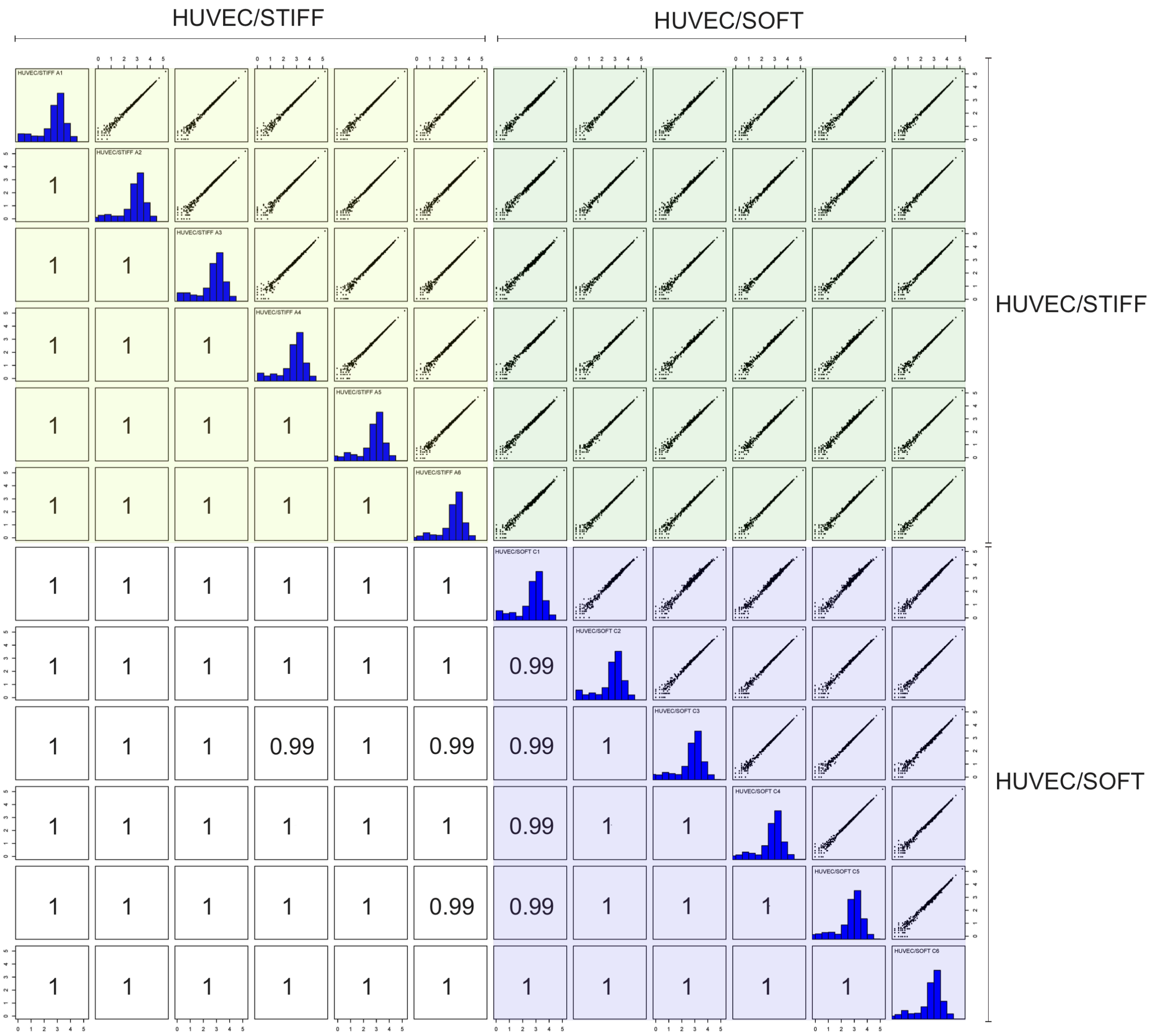

Supplementary Figure S3. Comparison of all samples of HUVEC on stiff matrices with HUVEC on soft matrices. All2All (see All2All function of Deseq2) scatter plots showing one by one comparison of the logarithm of normalized counts (x and y axes) of each sample with any other sample of the groups to be compared. The specific groups that are compared are HUVEC residing on stiff 70 kPa (N=6, yellow) to HUVEC residing on soft 3 kPa (N=6, blue) matrices. The blue histograms depict the distribution of gene expression for the corresponding samples. The values inside the white boxes indicate the R-squared values for the line of best fit for the corresponding diagonal scatter plots.

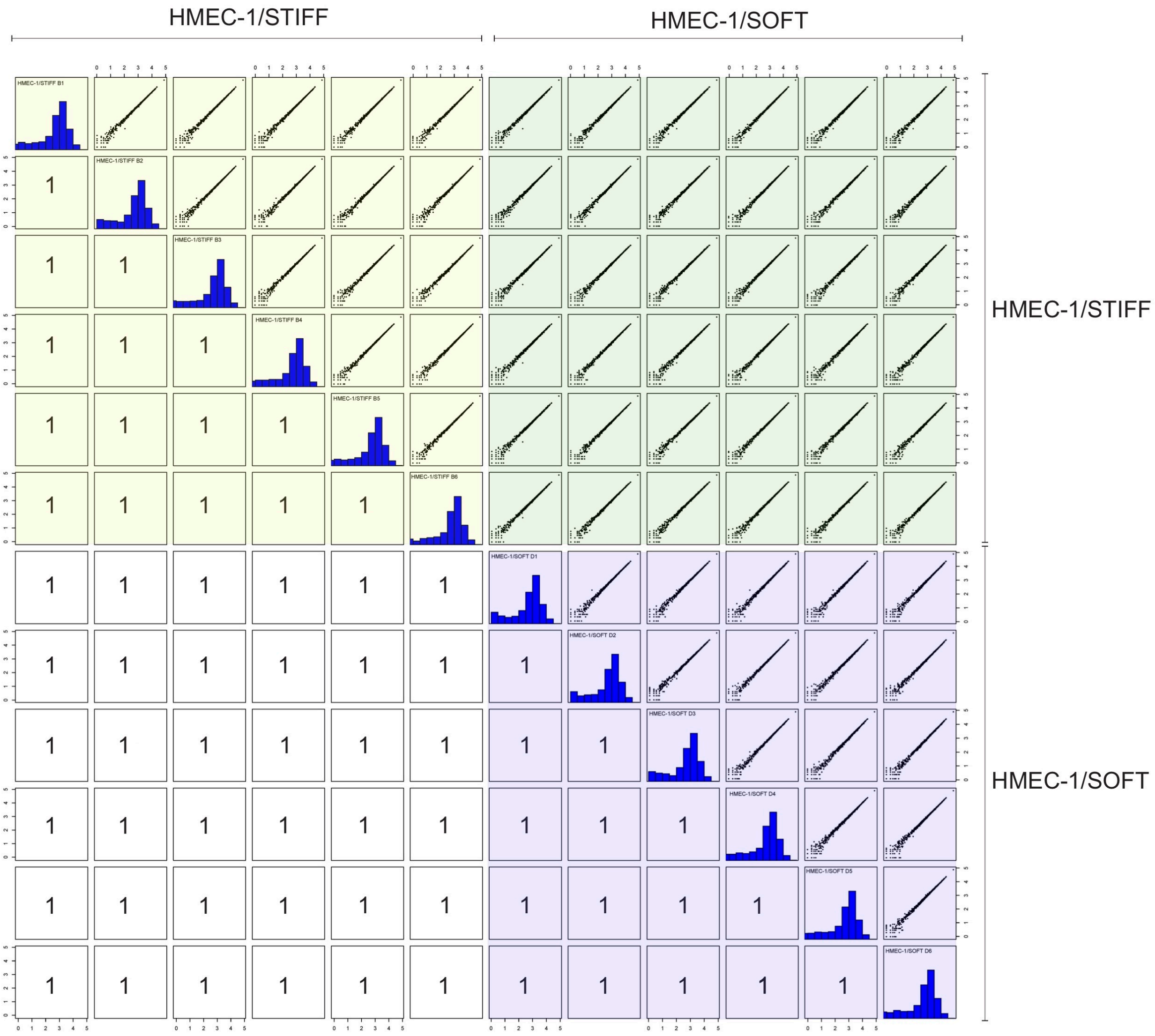

Supplementary Figure S4. Comparison of all samples of HMEC-1 on stiff matrices with HMEC-1 on soft matrices. Same as Fig. S1 but the specific groups that are compared are HMEC-1 residing on stiff 70 kPa matrices (N=6) to HMEC-1 residing on soft 3 kPa matrices (N=6).

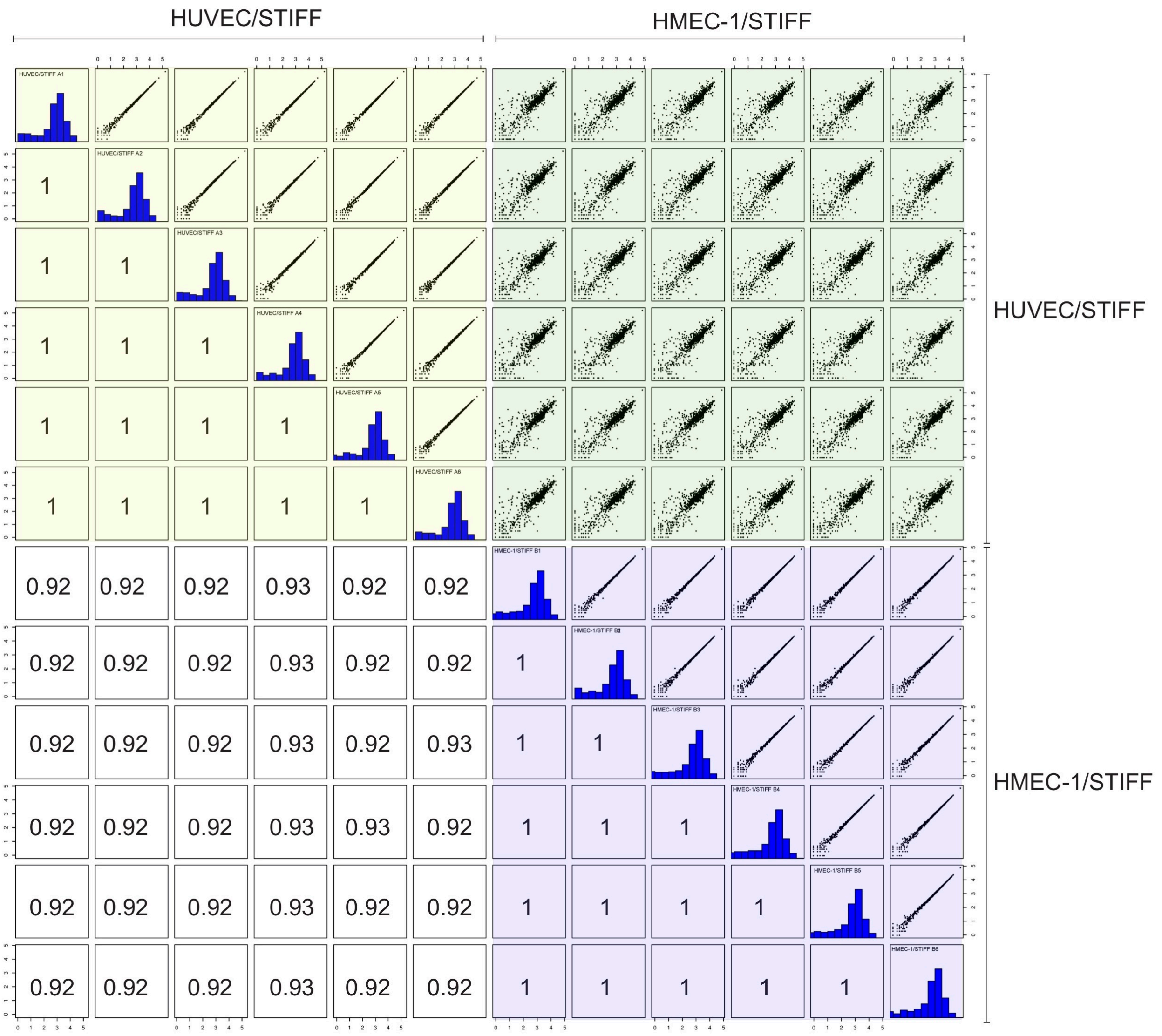

Supplementary Figure S5. Comparison of all samples of HUVEC on stiff matrices with HMEC-1 on stiff matrices. Same as Fig. S1 but the specific groups that are compared are HUVEC residing on stiff 70 kPa matrices (N=6) to HMEC-1 residing on stiff 70 kPa matrices (N=6).

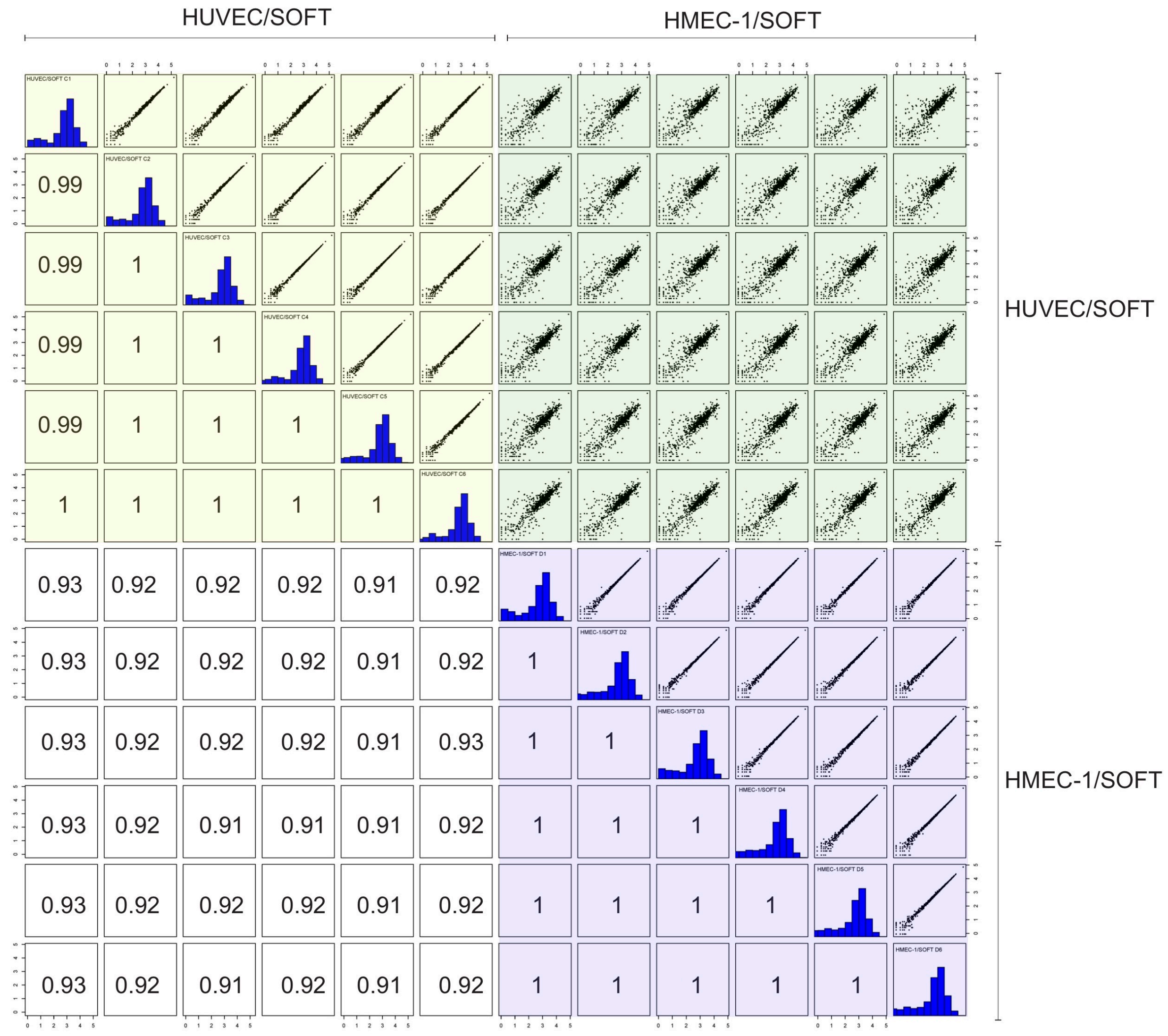

Supplementary Figure S6. Comparison of all samples of HUVEC on soft matrices with HMEC-1 on soft matrices. Same as Fig. S1 but the specific groups that are compared are HUVEC residing on soft 3 kPa matrices (N=6) to HMEC-1 residing on soft 3 kPa matrices (N=6).

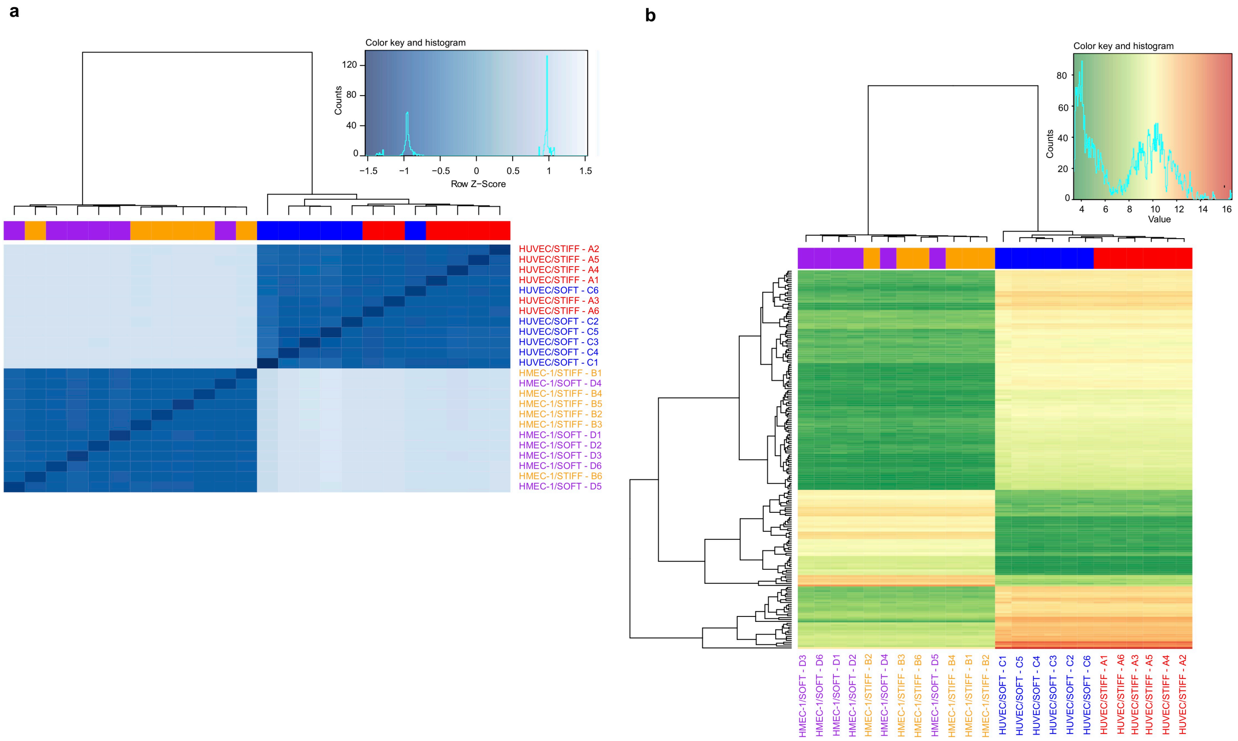

Supplementary Figure S7. RNA-sequencing sample distance (correlation) analysis and hierarchical clustering based on gene expression analysis. (**a**) RNA-sequencing count tables were statistically analyzed with DESEq2 and the Euclidean distances were calculated between each sample based on the *rlog* transformed data. The 24 samples were clustered using hierarchical clustering analysis and the dendrograms represent the clustering results. The heatmap shows the pairwise distances between the indicated samples according to the distance key shown on the upper right corner of the table. Distance table shows HUVEC on stiff 70 kPa (N=6, red), HUVEC on soft 3 kPa (N=6, blue), HMEC-1 on stiff 70 kPa (N=6, orange), HMEC-1 on soft 3 kPa matrices (N=6 purple). (**b**) Hierarchical cluster analysis of gene expression profiles based on top 200 genes with the most variance across all 24 samples indicated with different colors depending on the condition. Dendrograms are added on the top and left sides of the heatmap and reordered based on row means. Rows represent genes and columns individual samples from all four conditions as in panel A. Heatmaps are constructed based on the *rlog* transformed counts without being scaled (heatmap.2() function of DESeq2, see Table S6). Blocks of genes that covary are shown.

KEGG functional annotation between HUVEC (upregulated-green) versus HMEC-1 (upregulated-red) on stiff matrices

	p.geomean	stat.mean	p.val	q.val	set.size
**1. hsa04514 Cell adhesion molecules (CAMs)**	3.24814678490035E-08	5.59370783188084	3.24814678490034E-08	5.32696072723655E-06	130
**2. hsa00590 Arachidonic acid metabolism**	7.14858243623586E-05	3.95933817938119	7.14858243623588E-05	0.00586183759771342	59

1

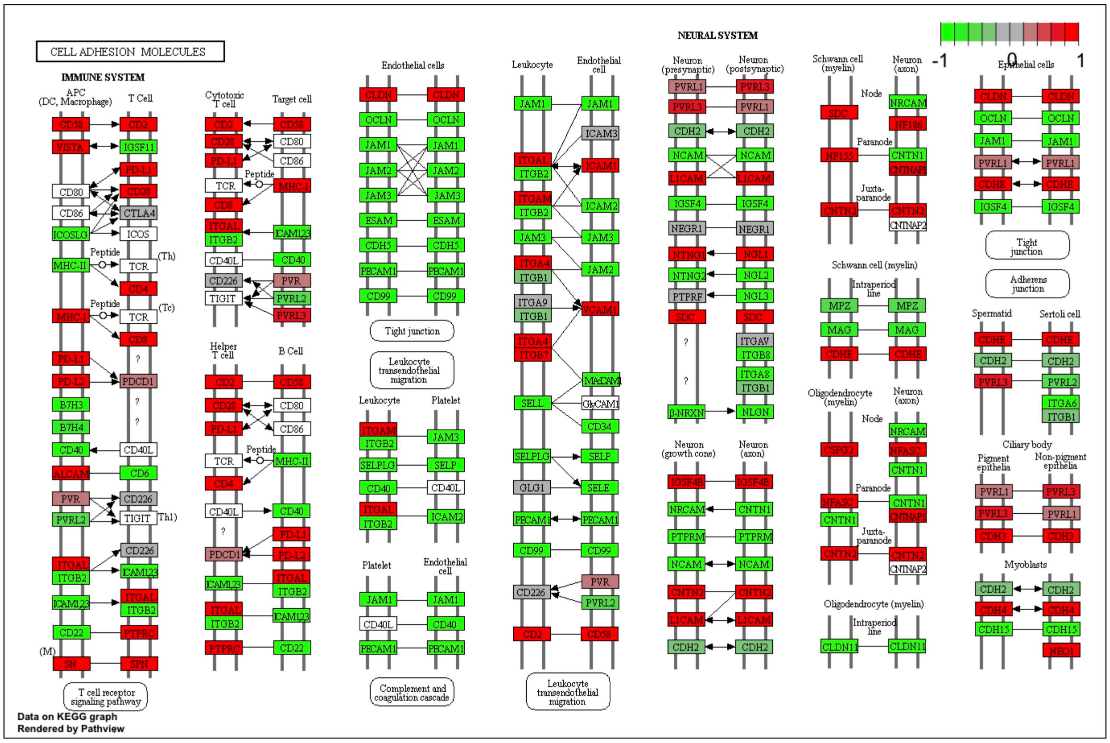

2

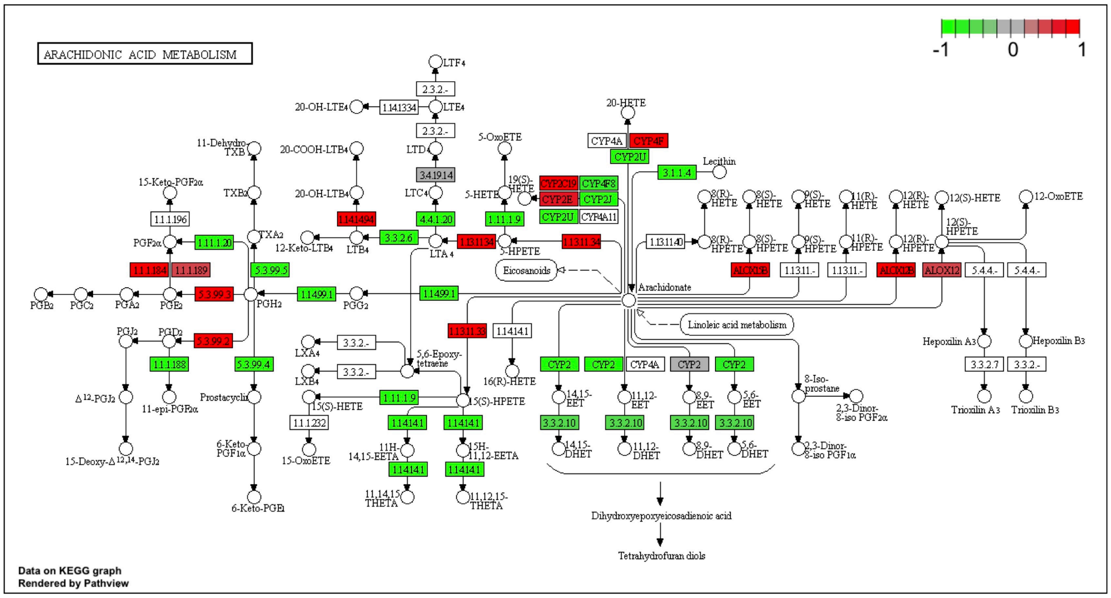

Supplementary Figure S8. Functional pathways differing for HUVEC as compared to HMEC-1. Signaling pathway visualization maps showing two of the signaling pathways that are differentially regulated for HUVEC residing on stiff matrices versus HMEC-1, namely: 1. Cell

adhesion molecules and 2. Arachidonic acid metabolism. The GAGE package was used for pathway analysis since it has precompiled databases for mapping genes to KEGG pathways[1-3]. The PATHVIEW package was used for visualization of the pathways. Detected DEGs are shown with colors according to their normalized enrichment score. Negative fold changes are shown in green and suggest that HUVEC gene expression is up whereas positive fold changes are shown in red and suggest that HMEC-1 gene expression is up.

KEGG functional annotation between HUVEC (upregulated-green) versus HMEC-1 (upregulated-red) on stiff matrices

	p.geomean	stat.mean	p.val	q.val	set.size
**5. hsa04512 ECM-receptor interaction**	0.000585534791057349	3.31816386243887	0.000585534791057351	0.0170007901342529	85
**7. hsa04020 Calcium signaling pathway**	0.000725643481340064	3.21035560232848	0.000725643481340063	0.0170007901342529	177

5

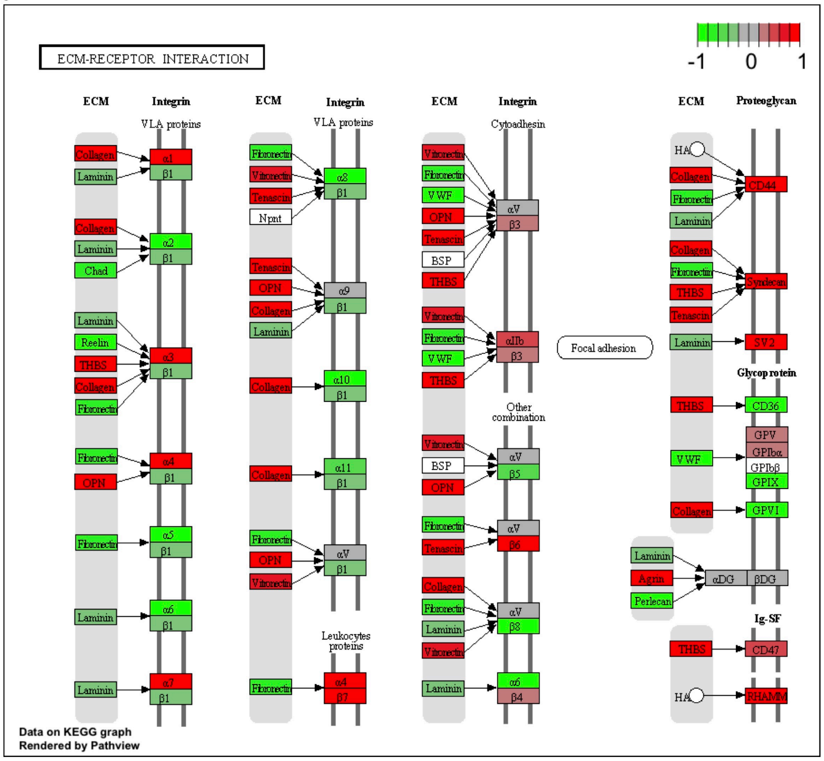

7

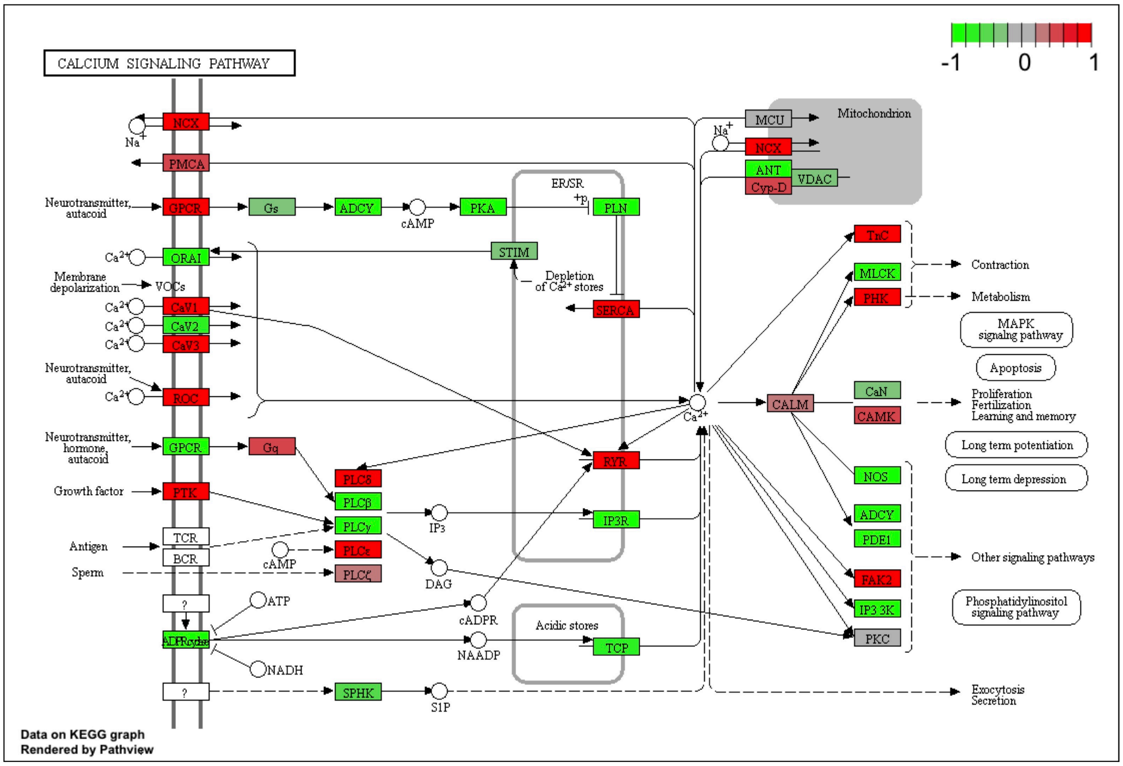

Supplementary Figure S9. Functional pathways differing for HUVEC as compared to HMEC-1. Signaling pathway visualization maps[1-3] showing two of the signaling pathways that are differentially regulated for HUVEC residing on stiff matrices versus HMEC-1, namely: 1. ECM-receptor interactions and 2. Calcium Signaling Pathway. The gage package was used for pathway analysis which has precompiled databases for mapping genes to KEGG pathways. Detected differentially expressed genes are shown with colors according to their normalized enrichment score. Negative fold changes are shown in green and suggest that HUVEC gene expression is up whereas positive fold changes are shown in red and suggest that HMEC-1 gene expression is up.

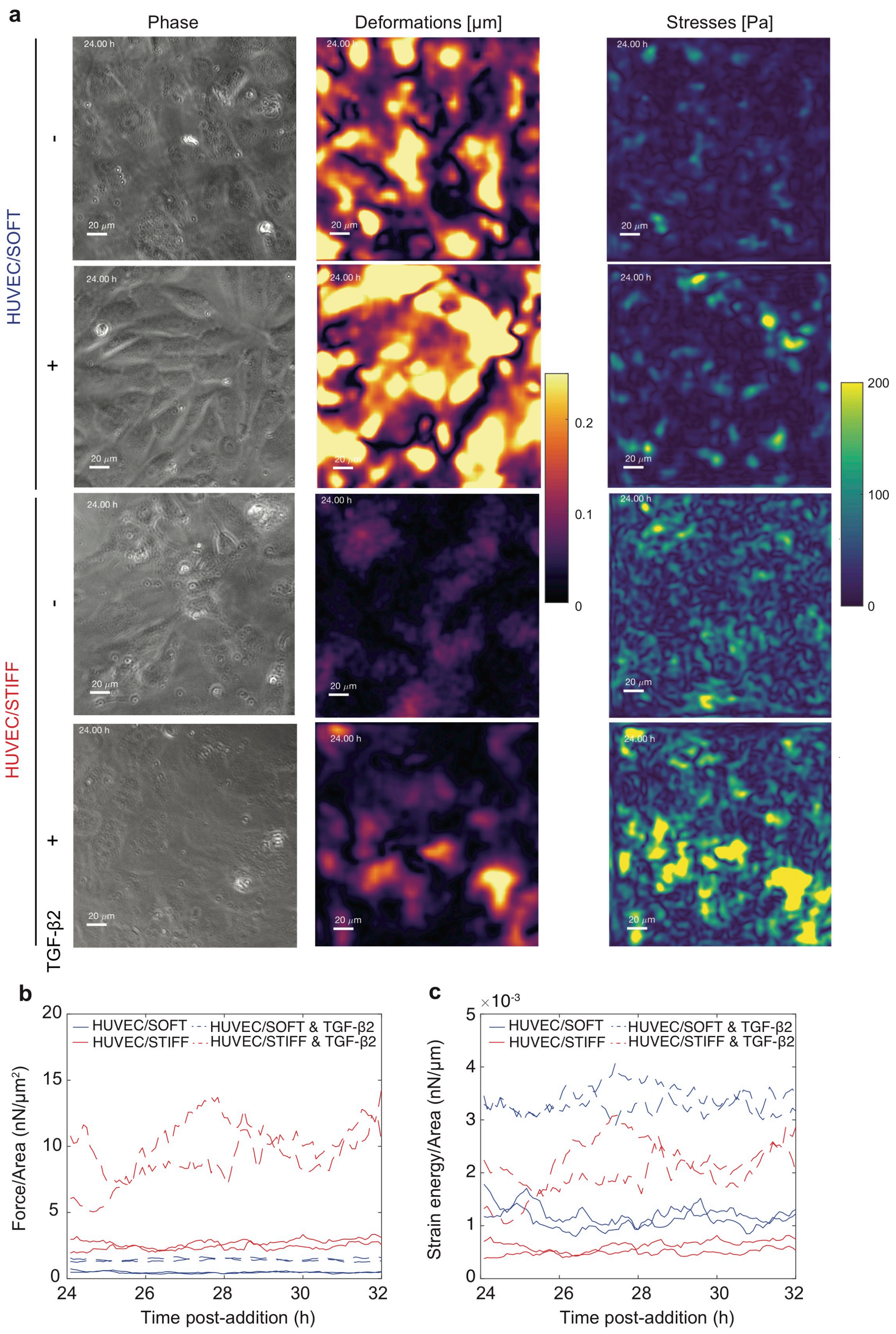

a
Phase
Deformations [μm]
Stresses [Pa]
HUVEC/SOFT
HUVEC/STIFF
-
+
TGF-β2
24.00 h
20 μm
0.2
0.1
0
200
100
b
HUVEC/SOFT
HUVEC/STIFF
HUVEC/SOFT & TGF-β2
HUVEC/STIFF & TGF-β2
Force/Area (nN/μm²)
Time post-addition (h)
c
×10⁻³
Strain energy/Area (nN/μm)
Time post-addition (h)

Supplementary Figure S10. Upregulation of HUVEC cell-matrix traction stresses 24 h after addition of TGF-β2. (**a**) Representative phase contrast images (first column) and maps showing the magnitude of cell-matrix deformation (second column, color indicates deformation magnitude in μm) and traction stresses (third column, color indicates stress magnitude in Pa) exerted by confluent HUVEC monolayers adherent onto soft 3 kPa (first and second rows) or stiff 35 kPa (third and fourth rows) hydrogels coated with collagen I and treated or not with 1 ng/mL TGF-β2. Images refer to t= 24 h post addition of TGF-β2 or vehicle control. (**b-c**) Time evolution of the integral of the traction force magnitude over the whole field of view to its area (nN/μm^2) (b) and of the total strain energy imparted by the cells per area of field of view (nN/μm) (c) calculated for two different regions within confluent HUVEC monolayers for cells residing on soft 3 kPa (blue) or stiff 35 kPa (red) matrices. Cells were either treated with vehicle control (solid line) or 1 ng/mL TGF-β2 (dashed line) for 24 h prior to imaging.

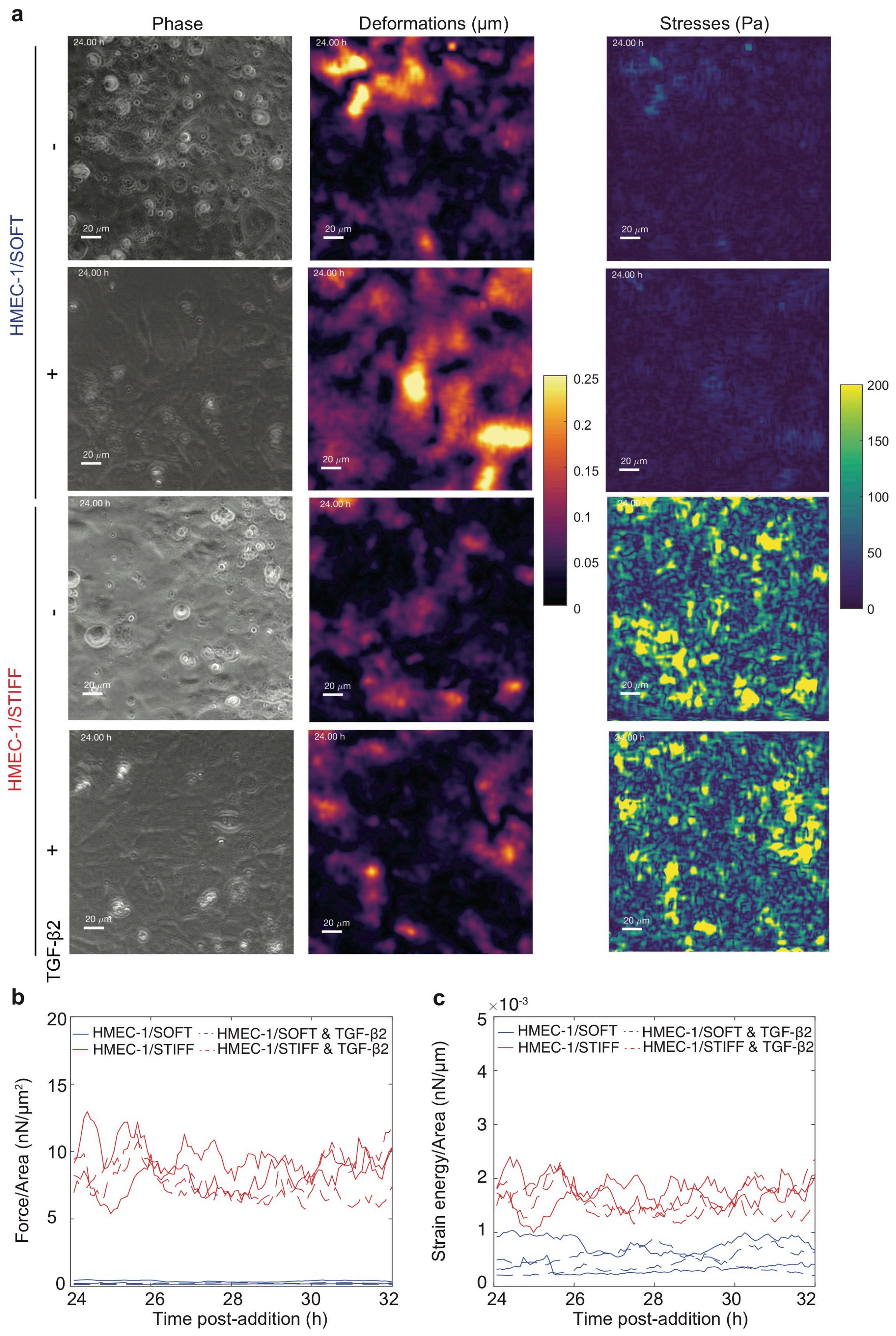
a
Phase
Deformations (μm)
Stresses (Pa)
HMEC-1/SOFT
HMEC-1/STIFF
-
+
TGF-β2
24.00 h
20 μm
0.25
0.2
0.15
0.1
0.05
0
200
150
100
50
0
b
HMEC-1/SOFT
HMEC-1/STIFF
HMEC-1/SOFT & TGF-β2
HMEC-1/STIFF & TGF-β2
Force/Area (nN/μm²)
20
15
10
5
0
24
26
28
30
32
Time post-addition (h)
c
×10-3
HMEC-1/SOFT
HMEC-1/STIFF
HMEC-1/SOFT & TGF-β2
HMEC-1/STIFF & TGF-β2
Strain energy/Area (nN/μm)
5
4
3
2
1
0
24
26
28
30
32
Time post-addition (h)

Supplementary Figure S11. No increase in HMEC-1 cell-matrix traction stresses 24 h after addition of TGF-β2. (**a**) Representative phase images (first column) and maps showing the magnitude of cell-matrix deformation (second column, color indicates deformation magnitude in μm) and traction stresses (third column, color indicates stress magnitude in Pa) exerted by confluent HMEC-1 monolayers adherent onto soft 3 kPa (first and second rows) or stiff 35 kPa (third and fourth rows) hydrogels coated with collagen I and treated or not with 1 ng/mL TGF-β2. Images refer to t= 24 h post addition of TGF-β2 or vehicle control. (**b-c**) Time evolution of the integral of the traction force magnitude over the whole field of view to its area (nN/μm^2) (b) and of the total strain energy imparted by the cells per area of field of view (nN/μm) (c) calculated for two different regions within confluent HMEC-1 monolayers for cells residing on soft 3 kPa (blue) or stiff 35 kPa (red) matrices. Cells were either treated with vehicle control (solid line) or 1 ng/mL TGF-β2 (dashed line) for 24 h prior to imaging.

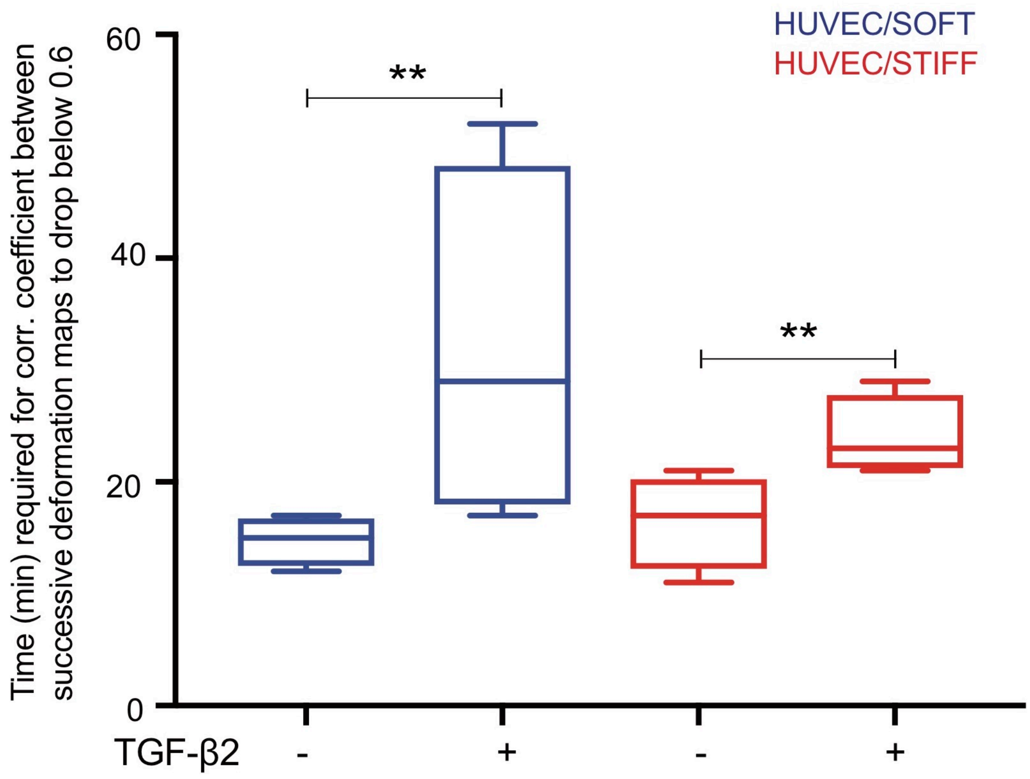

Supplementary Figure S12. Slowing of dynamics of HUVEC traction adhesions upon addition of TGF-β2. Boxplots of the time needed for the correlation coefficient between successive in time deformation maps produced by HUVEC monolayers to fall below 0.6. HUVEC resided either on soft 3 kPa or stiff 35 kPa matrices and we also either treated with vehicle control or 1 ng/mL TGF-β2 for 24 h prior to imaging. The deformation map of each frame was correlated to the deformation map of up to 12 frames later (Δframe= 10 min) and the resulting average correlation coefficients depending on time separation were calculated. For each experiment the mean time necessary for the correlation coefficient to drop to 0.6 was identified and shown for HUVEC on soft 3 kPa (blue) or stiff 35 kPa (red) matrices when cells were treated with vehicle control or 1 ng/mL TGF-β2 for 24 h (N = 4 experiments).

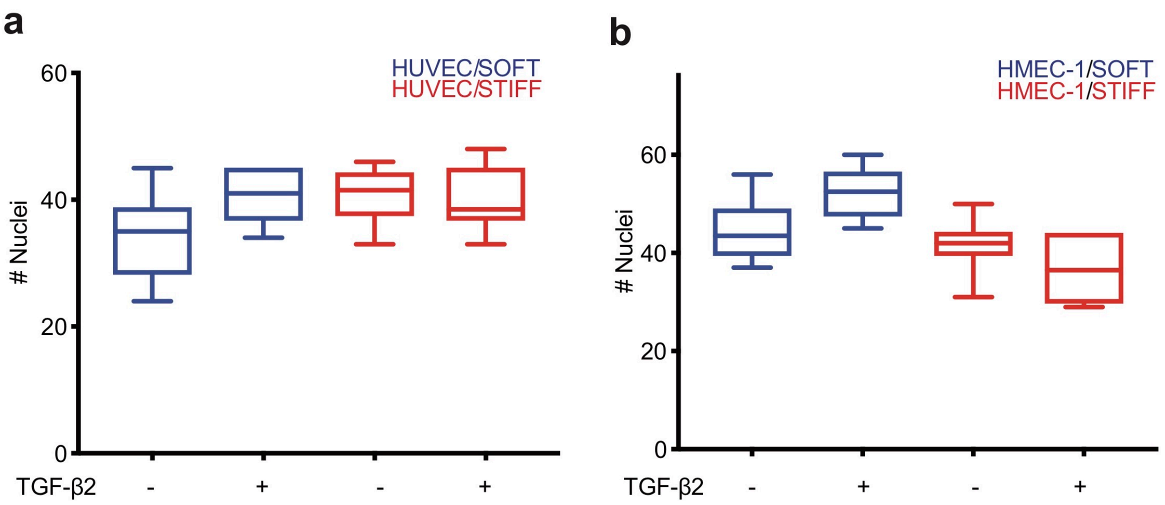

Supplementary Figure S13. Similar nuclei density for HUVEC and HMEC on soft and stiff hydrogels. (**a**) Boxplots of the number of nuclei per field of view for HUVEC monolayers treated or not with 1 ng/mL TGF-β2 for 24 h and residing on soft 3 kPa or stiff 70 kPa hydrogels. Cells' nuclei were stained with DAPI. Corresponds to same images as those used to produce boxplots in Fig. 6Q-R. (**b**) Boxplots of the number of nuclei per field of view for HMEC-1 monolayers treated or not with 1 ng/mL TGF-β2 for 24 h and residing on soft 3 kPa or stiff 70 kPa hydrogels. Cells' nuclei were stained with DAPI. Corresponds to same images as those used to produce boxplots in Fig. 6e-h.

SUPPLEMENTARY TABLE CAPTIONS

Supplementary Table S1. DEGs between HUVEC residing on soft 3 kPa versus stiff 70 kPa hydrogels. Table presents the DEGs identified when comparing the transcriptome of HUVEC monolayers residing on soft 3 kPa versus stiff 70 kPa hydrogels (N = 6 samples per condition). Rows correspond to the genes identified and columns B-J correspond to the Ensemble IDs of the genes (ENSID), the normalized mean of all counts (baseMean), the $\log_2$ fold change in gene expression between groups (lfc), the standard deviation of the $\log_2$ fold change (lfcSE), the wald statistic defined as the ratio of lfc to lfcSE (stat), the p-value (pvalue), the adjusted for multiple testing p-value (padj), the symbol of the specific gene (symbol) and the actual name of the gene (name). Columns J-O and P-U provide the normalized counts of each sample from the corresponding groups compared. Rows are ordered based on the wald statistic with highly negative values corresponding to genes upregulated for HUVEC on stiff matrices.

Supplementary Table S2. DEGs between HMEC-1 residing on soft 3 kPa versus stiff 70 kPa hydrogels. Same as Suppl. Table I but presenting the DEGs identified when comparing the transcriptome of HMEC-1 monolayers residing on soft 3 kPa versus stiff 70 kPa hydrogels (N = 6 samples per condition). Rows are ordered based on the wald statistic with highly negative values corresponding to genes upregulated for HMEC-1 on stiff matrices.

Supplementary Table S3. DEGs between HUVEC versus HMEC-1 residing on stiff 70 kPa hydrogels. Same as Table I but presenting the DEGs identified when comparing the transcriptome of HUVEC versus HMEC-1 monolayers residing on stiff 70 kPa hydrogels (N = 6 samples per condition). Rows are ordered based on the wald statistic with highly negative values corresponding to genes upregulated for HUVEC.

Supplementary Table S4. DEGs between HUVEC versus HMEC-1 residing on soft 3 kPa hydrogels. Same as Table I but presenting the DEGs identified when comparing the transcriptome of HUVEC versus HMEC-1 monolayers residing on soft 3 kPa hydrogels (N = 6 samples per condition). Rows are ordered based on the wald statistic with highly negative values corresponding to genes upregulated for HUVEC.

Supplementary Table S5. Genes expressed only in HMEC-1 or in HUVEC. Table showing the Ensemble ID (ENSID) and symbol of DEGs found to be expressed only in HMEC-1 (columns B and C) or only in HUVEC (columns E and F).

Supplementary Table S6. Top 200 genes with most variance across all 24 samples. Hierarchical cluster analysis of gene expression profiles based on the top 200 genes with the most variance across all 24 samples was performed (see Figure SVB). The resulting top variant genes are shown in each row. Columns show their Ensemble ID (ENSID), the *rlog* transformed counts of each sample and the symbol of the corresponding gene.

Supplementary Table S7. 9 KEGG pathways are significantly perturbed when comparing HUVEC to HMEC-1. The gage package was used for pathway analysis which has precompiled databases for mapping genes to KEGG pathways so to perform pathway enrichment analysis of DEGs between HUVEC versus HMEC-1 residing on stiff matrices. 9 KEGG pathways were identified to be significantly perturbed when comparing HUVEC to HMEC-1 (padj<0.05). Rows show the KEGG pathways and column show the geometrical mean of the individual p-values from multiple single array based gene set tests (p.geomean), the mean of the individual statistics from multiple single array based gene set tests (stat.mean), the summary of the individual p-values from multiple single array based gene set tests (p.value), the false discovery rate adjusted global p-value (padj) and the number of genes included in the gene set test (set.size).

Supplementary Table S8. GO Biological Processes (BP) ontologies that are significantly perturbed when comparing HUVEC to HMEC-1. The GAGE package was used for pathway analysis which has precompiled databases for mapping genes to GO BP terms (see Materials and Methods). GO BP enrichment analysis was performed to compare HUVEC versus HMEC-1 residing on stiff matrices. 11 BPs were found to be upregulated for HMEC-1 (rows 1-12) and 46 BPs were upregulated for HUVEC (rows 15-61) when comparing HUVEC to HMEC-1 (padj<0.01). Rows show the GO BPs that are significantly up- or down- regulated and columns show the geometrical mean of the individual p-values from multiple single array based gene set tests (p.geomean), the mean of the individual statistics from multiple single array based gene set tests (stat.mean), the summary of the individual p-values from multiple single array based gene set tests (p.value), the false discovery rate adjusted global p-value (padj) and the number of genes included in the gene set test (set.size).

Supplementary Table S9 RT-PCR primers. List of the RT-PCR primers used for this study.

SUPPLEMENTARY MOVIE CAPTIONS

Supplementary Movie S1. Deformation and cell-matrix traction stresses exerted by HUVEC on soft 3 kPa matrices after addition (or not) of TGF-β2. Movie shows the deformation map (left column) and traction stress map (right column) that confluent HUVEC produce while residing on soft 3 kPa matrix. Cells resided on these matrices for 24 h and were then treated with vehicle control (upper row) or 1 ng/mL TGF-β2 for 24 h prior to imaging (bottom row). Time post-addition of vehicle control or is TGF-β2 indicated.

Supplementary Movie S2. Deformation and cell-matrix traction stresses exerted by HUVEC on stiff 35 kPa matrices after addition (or not) of TGF-β2. Movie shows the deformation map (left panel) and traction stress map (right panel) that confluent HUVEC produce while residing on stiff 35 kPa kPa matrix. Cells resided on these matrices for 24 h and were then treated with vehicle control (upper row) or 1 ng/mL TGF-β2 for 24 h prior to imaging (bottom row). Time post-addition of vehicle control or is TGF-β2 indicated.

SUPPLEMENTARY REFERENCES

1 Kanehisa, M. & Goto, S. KEGG: kyoto encyclopedia of genes and genomes. *Nucleic acids research* **28**, 27-30, doi:10.1093/nar/28.1.27 (2000).

2 Kanehisa, M., Sato, Y., Furumichi, M., Morishima, K. & Tanabe, M. New approach for understanding genome variations in KEGG. *Nucleic Acids Res* **47**, D590-d595, doi:10.1093/nar/gky962 (2019).

3 Kanehisa, M. Toward understanding the origin and evolution of cellular organisms. *Protein Sci* **28**, 1947-1951, doi:10.1002/pro.3715 (2019).
